# Proteome analysis of male accessory gland secretions in oriental fruit flies reveals juvenile hormone-binding protein, suggesting impact on female reproduction

**DOI:** 10.1038/srep16845

**Published:** 2015-11-19

**Authors:** Dong Wei, Hui-Min Li, Chuan-Bei Tian, Guy Smagghe, Fu-Xian Jia, Hong-Bo Jiang, Wei Dou, Jin-Jun Wang

**Affiliations:** 1 Key Laboratory of Entomology and Pest Control Engineering, College of Plant Protection, Southwest University, Chongqing 400716, China; 2 Department of Crop Protection, Ghent University, B-9000 Ghent, Belgium

## Abstract

In insects, the accessory gland proteins (Acps) secreted by male accessory glands (MAGs) account for the majority of seminal fluids proteins. Mixed with sperm, they are transferred to the female at mating and so impact reproduction. In this project, we identified 2,927 proteins in the MAG secretions of the oriental fruit fly *Bactrocera dorsalis*, an important agricultural pest worldwide, using LC-MS analysis, and all sequences containing open reading frames were analyzed using signalP. In total, 90 Acps were identified. About one third (26) of these 90 Acps had a specific functional description, while the other two thirds (64) had no functional description including dozens of new classes of proteins. Hence, several of these novel Acps were abundant in the MAG secretions, and we confirmed their MAG-specific expression by qPCR. Finally and interestingly, one of these novel proteins was functionally predicted as juvenile hormone-binding protein, suggesting the impact of Acps with reproductive events in the female. Our results will aid in the development of an experimental method to identify Acps in insects, and in turn this information with new Acps in *B. dorsalis* will pave the way of further exploration their function in reproduction and potential development as new insecticide targets.

The male accessory glands (MAGs) produce a variety of secreted proteins and peptides that are known as accessory gland proteins (Acps), which are transferred from the male to the female, together with sperm during copulation. These Acps play a critical role in insect fertilization, with the function not limited to males^1^. Their influence also extends to females, including modulating the outcome of postmating sexual selection, female reproductive physiology and immune responses^2^. Acps can also affect sperm storage parameters, sperm competition and mating plug formation^1^,^2^. Most of these changes in females result from gene expression changes induced by Acps^3^,^4^. Early study of reproductive proteins began when the biochemical fractionation of gametes and gonads led to the purification and characterization of specific proteins. Recently, various molecular and genetic tools, coupled with bioinformatics, have been widely used to identify and analyze Acps in many insects^1^,^5^. However, these proteins are not well characterized outside the *Drosophila* species, although some works have been done in grasshoppers, mosquitoes, moths, beetles, crickets, and honeybees^5^,^6^,^7^,^8^. Recent efforts have been made to extend our knowledge of reproductive protein evolution across species that are not well characterized genetically. These studies have used proteomic approaches to identify and sequence proteins derived from reproductive accessory glands and/or directly from the seminal fluid of *Drosophila* species^6^. Such approaches have also successfully identified reproductive proteins from the ejaculates of non-model species^5^,^9^,^10^,^11^,^12^,^13^.

Proteomic analysis can identify thousands of proteins in complex biological samples^14^. In recent years, with the aid of proteomic approaches, comprehensive studies have been initiated to identify and analyze the Acps in many insect species for which we are lacking genomic information. There has also been an increase in the number of studies on insect MAGs and testes that investigate the function of specific seminal proteins through transcriptomic^15^,^16^ and proteomic analysis^11^,^17^. Mass spectrometry can be used to characterize a set of expressed proteins, and has been used previously in the identification and evolutionary analysis of Acps^18^. In *Heliconius* butterfly MAGs, 51 Acps have been identified by a combination of EST and proteome analyses, and these included chymotrypsin, proteinase inhibitor and hormone binding protein^12^. These secreted Acps showed a high presence in the MAGs, and are good candidates that may be transferred to the female during copulation. In *Drosophila*, there were also 138 seminal fluid proteins that were identified, including some new seminal fluid proteins named Obp56f, Obp56g and CG17472^6^.

The oriental fruit fly *Bactrocera dorsalis* is an important agricultural pest worldwide^19^. To date, information is lacking on the Acps of *B. dorsalis* because of difficulties identifying the individual proteins. Proteomics is a novel and direct method to identify unannotated Acps in insect MAGs. In a previous study, we obtained a tissue-specific RNA sequencing database of the MAGs of *B. dorsalis*^20^. The latter data are useful so to identify the Acps via similarity searching, which method was also done previously for Acps identification in butterflies^12^. In the present study, we carried out liquid chromatogram mass spectrometry (LC-MS) analysis of the proteins secreted by the MAGs of *B. dorsalis*. This combined transcriptomic and proteomic analysis then allowed to identify and characterize the Acps with the help of open reading frame (ORF) and signal peptide prediction. Among the novel Acps identified in male *B. dorsalis*, we found one interesting that was functionally predicted as juvenile hormone-binding protein (JHBP), suggesting the impact of Acps with the reproductive event in the female adult.

## Results

### Proteomic analysis of proteins secreted by the MAGs

**Figure 1:**
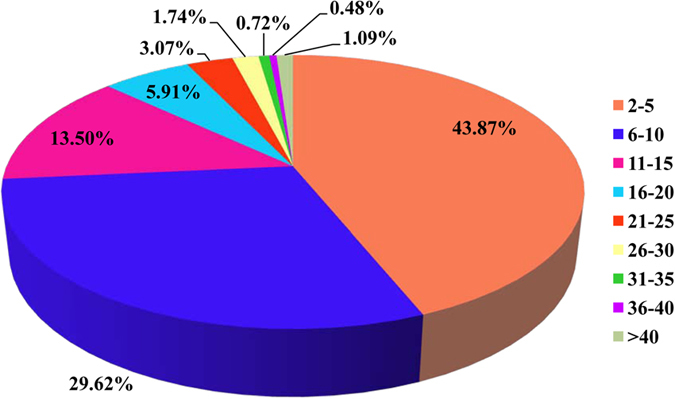
The distribution of identified peptides of proteins by proteomic analysis of the secretions of the MAGs from *B. dorsalis* as identified by mass spectrometry.

**Figure 2:**
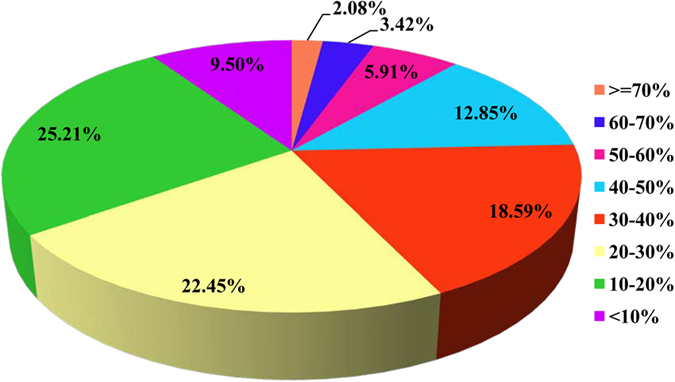
Proteomic analysis of proteins in the secretions of the MAGs from *B. dorsalis* as identified by mass spectrometry.

**Figure 3:**
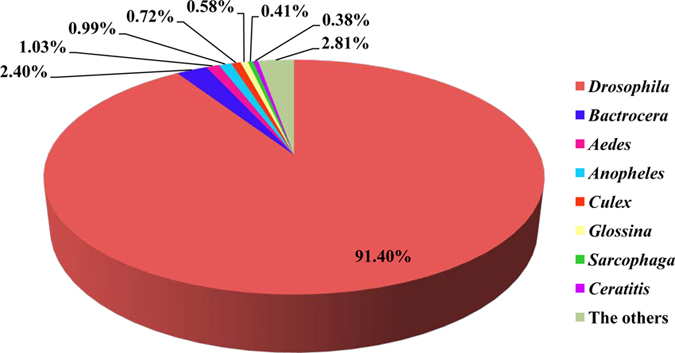
Homology analysis of identified CDSs/proteins in the secretions of the MAGs from *B. dorsalis* as identified by mass spectrometry.

A total of 30,669 unigenes were assembled by this transcriptomic sequencing^20^. The assembled sequences were thereafter deposited at DDBJ/EMBL/GenBank with the Transcriptome Shotgun Assembly project accession number of GDRP00000000. After proteins coding sequences (CDSs) prediction analysis in transcriptome data, 19,484 unigene sequences were assembled to match known proteins following BLASTx analysis of protein databases. Sequences with an amino acid sequence match were designated as CDSs. In total, most of the CDSs were shorter than 500 residues. The Acps secreted by MAGs were separated and identified by label-free LC-MS based on the CDSs dataset. In total, 27,038 and 27,702 peptides were detected in each sample, respectively, which corresponded to 3,527 and 3,984 proteins in each sample, respectively. The raw data of the current proteomics were available from the authors upon request. After combining all data from the two samples, we identified 25,606 peptides that were present in both samples. These peptides were matched to 3,942 protein groups, for which we were able to annotate 2,927 significant CDSs. Notably, there were no proteins that were identified by only one peptide, and most of these proteins corresponded to between two and ten peptides (73.5%, Fig. 1). Thirty-two proteins were identified by more than forty peptides. The average number of peptides per protein was 8.75, leading to an average sequence coverage of 29.2 times (Fig. 2). There were 334 proteins (11.4%) with a sequence coverage of >50%. Because *B. dorsalis* is not a model organism and there is no complete genome sequence available currently, the species homology analysis was performed. Almost all of the proteins showed the greatest homology to Diptera species, of which 91.2% were *Drosophila*, followed by *Bactrocera* (2.39%), *Aedes* (1.02%) and other genera (5.89%) (Fig. 3).

### Acps identification in the MAGs

Most of the proteins had no specific annotation or functional description in the databases. Following ORF prediction, 1,116 proteins (40.9%) containing ORFs were screened out. In most cases, extracellularly secreted proteins were identified by the presence of a signal peptide. Using SignalP, 90 of these ORFs contained predicted 5′ signal peptides that were identified as putative Acps (Table 1 and Supplementary Data). Amongst these there were 26 known proteins and 64 proteins with no functional description (>70%). The known proteins consisted of proteases, odorant binding proteins (Obps), metalloproteinase, ribosomal protein, serine protease inhibitor, and some immunity-related proteins.

### Most abundant Acps in the MAGs

**Figure 4:**
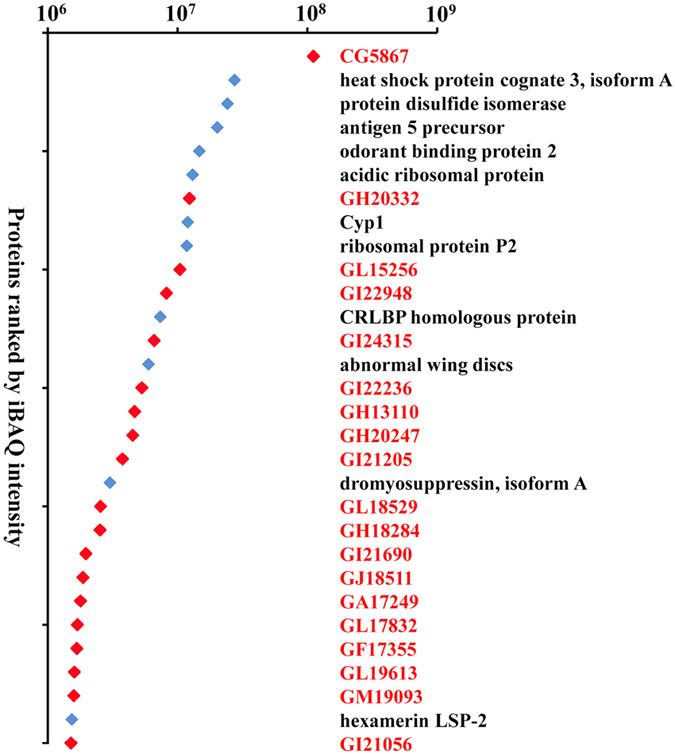
Selection of the 30 most abundant proteins in the secretions of the MAGs from *B. dorsalis*. The total absolute expression value of each protein in two samples was estimated by intensity-based absolute quantification (iBAQ). Red labels indicate novel Acps with no functional description.

We estimated the relative molar abundance of the predicted Acps in the MAGs secretions using label-free MS and the intensity-based absolute quantification (iBAQ) algorithm. Most of the abundant proteins were of unknown function, which was consistent with the protein content. Of the 30 most abundant Acps, 19 were unknown proteins (Fig. 4). The true contribution of a particular protein to the total mass of the secretion is the product of its molar abundance and its molecular weight. Using this determination, the most abundant protein was CG5867, a protein of unknown function, with a molar proportion of 4.38% of total protein. Several of most abundant proteins were previously-characterized Acps, such as Obp21 and the immune-related proteins, cyclophilins. However, several proteins that were previously not linked to MAG secretions, were also in the top quartile for abundance, including the most abundant six protein CG5867, GH20332, GL15256, GI2948, GI24315 and GI22236, etc.

### Tissue-specific expression at mRNA level and functional prediction of novel Acps from the MAGs

The majority of Acps identified in this study showed no significant BLASTx similarity to proteins in GenBank. After combined InterProScan and Gene Ontology analysis, there were 58 Acps were functional predicted. All of these predicted proteins were classified into 11 categories based on their molecular functions including 32 unknown proteins (Table 2). Proteases, protease inhibitors, mediators of immune responses, and odorant binding included more Acps than the other categories. There were also 32 (35.6%) proteins of unknown function.

**Figure 5:**
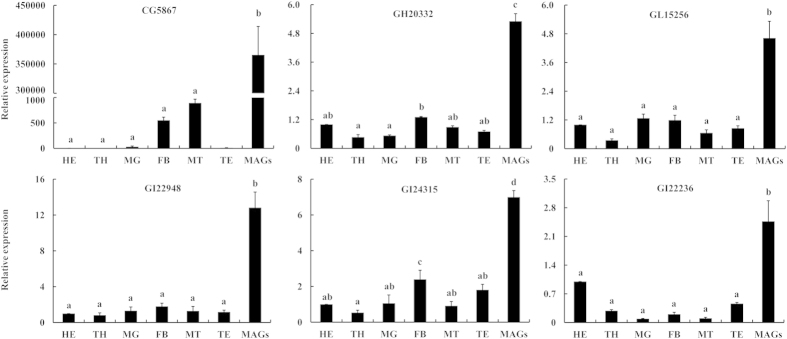
Quantitative real time PCR validation of the high and tissue-specific expression of the six most abundant and novel Acps in the secretions of the MAGs from *B. dorsalis*. Relative expressions were determined in head (HE), thorax (TH), midgut (MG), fat body (FB), Malpighian tubules (MT), testis (TE), and male accessory glands (MAGs) from male *Bactrocera dorsalis*. Relative expression levels were calculated based on the value in head tissue, which was ascribed an arbitrary value of 1. Different letters above the bars indicate significant differences based on Tukey’s test (*P* < 0.05).

In the present proteomic analysis, we identified many Acps with best matches to not described proteins in the NCBI nr database. These proteins may be novel Acps to *B. dorsalis* flies. The six most abundant and novel Acps were assayed for tissue-specific expression patterns from standard concentrations of total RNA isolated from tissues of male *B. dorsalis* using real time PCR. Of these, all the determined six novel Acp genes were highly and specifically expressed in the MAGs of *B. dorsalis* (Fig. 5). The first and most interesting, *CG5867* was expressed in the MAGs with expression levels tens of thousands of times higher than in the head. The functional prediction indicated that it was a hemolymph juvenile hormone-binding protein (JHBP) by Blasting in InterProScan online tool. The second one GH20332, also highly expressed in MAGs, was predicted as cyclophilin-type peptidyl-prolyl cis-trans isomerase. Cyclophilins can exhibit peptidyl-prolyl cis-trans isomerase activity, accelerating protein folding by catalyzing the cis-trans isomerization of proline imidic peptide bonds in oligopeptides. This kind of cyclophilin also has protein chaperone-like functions. GL15256 had no functional prediction in NCBI and InterPro, but it showed a domain signature named kazal domain. The fourth most abundant, GI22948, was predicted as a mesencephalic astrocyte-derived neurotrophic factor homolog, and the fifth GI24315 as a heat shock protein 90 family protein. The last Acp-specific, GI22236, had no functional prediction in databases. We expect that these Acps that are highly and specifically present in the MAGs may be transferred to females to regulate the reproductive physiology of the insect.

## Discussion

Reproductive proteins maintain species-specific barriers to fertilization, affect the outcome of sperm competition, and mediate reproductive conflicts between the sexes^18^. However, specific proteins and molecular mechanisms that underlie these processes in *B. dorsalis* are virtually unknown. This is the first study to report the identification of Acps in *B. dorsalis*. The study provides a proteomic-scale view of the Acps secreted by *B. dorsalis* MAGs. Based on the MAG-specific transcriptome sequence data, 90 Acps were identified in the *B. dorsalis* MAGs. This project confirmed that a combination of transcriptomic analysis, proteomic analysis, bioinformatics and expression assays is an effective method for identifying Acps in organisms for which a reference genome sequence is not available. A recent explosion of proteomics studies has identified many reproductive proteins in organisms such as *Drosophila*^6^,^18^, *Heliconius* (butterfly)^12^,^21^, *Gryllus* (cricket)^22^, *Apis mellifera* (honeybee)^23^ and *Tribolium castaneum* (red flour beetle)^17^. In the accessible genomes of insect species, there is much more genetic information on reproductive proteins. For instance, in *Drosophila*, there were also 138 Acps that were identified by proteomic analysis in seminal fluids^6^. In these insects with genome information, there are also many orphan genes^24^,^25^. These genes have no recognizable homology to any sequences in other species. This is also one of the reasons that most of the Acps identified in the MAGs secretions had no functional prediction in the NCBI nr database. As shown here and previously, Acps are short, rapidly evolving, and relatively free of codon bias^26^,^27^. As such, Acps are less likely to be detected by computational gene prediction programs. Nonetheless, the Acps identification method used in this study was straightforward and high-efficiency for Acps identification. Recently, the genome of *B. dorsalis* has been sequenced and uploaded into the online database of NCBI (ftp://ftp.ncbi.nlm.nih.gov/genomes/Bactrocera_dorsalis/), but to date not much is known about the genes related to reproduction in this species.

There were 90 Acps that were identified in the secretions of the MAGs of *B. dorsalis*, and they were assigned to 11 categories based on their molecular functions. Most of these proteins had previously been identified as Acps in other insects, including proteases, protease inhibitors and ion binding proteins^6^,^12^. Almost 36% of these Acps were not identified as functional unknown proteins. In two previous studies of Acps in *D. melanogaster* and *Heliconius* butterflies, 34% and 60% of the proteins remained as unknown, although these insect have been studied well^6^,^12^. In this study, only sequences containing ORFs were considered as Acp candidates; however, it is likely that other Acps with no ORFs were also picked up by LC-MS. The availability of a whole genome sequence would increase the success of Acps identification. Other proteins such as transferrin, calmodulin and juvenile-related proteins, which were studied as Acps in other insects, were also identified although they were without ORFs^11^,^28^,^29^. Furthermore, not every protein identified in a mass spectrometry screen as a reproductive protein actually has a function in reproduction^30^, since some proteins may also play a housekeeping role that does not relate specifically to reproduction.

Acps are commonly abundant with proteases and protease inhibitors in many other taxa ranging from insects to mammals^2^,^31^. Proteases and protease inhibitors are also highly abundant in the sperm proteome of *Drosophila*^32^. Protease inhibitors are commonly identified as Acps in previous studies^11^,^18^, and are involved in sperm-egg interactions together with specific proteases^33^. Notably, cyclophilin and cyclophilin-type peptidyl-prolyl cis-trans isomerase were identified in the current study. These proteins are involved in the immune response during reproduction. The later one, GH20332, was identified as a novel Acp in the *B. dorsalis* MAGs secretions. Cyclophilins have been identified as Acps in *D. melanogaster*^34^, *Anopheles gambiae*^8^, *A. aegypti*^35^ and *L. longipalpis*^36^. Cyclophilins is one of the antimicrobial peptides (AMPs) that exist widely in both the male and female reproductive tracts of insects and mammals, and they are presumed to protect against microorganisms^37^,^38^. It has been demonstrated that male insects will transfer antibacterial proteins from their accessory glands and ejaculatory duct (ED) to their mates to increase their reproductive success^38^. Another immune protein, antigen 5, was also identified in the MAG/ED secretions of *B. dorsalis*. Antigen 5 is expressed mostly in MAGs (Wei D and Wang JJ, unpublished data) and is a major allergen of venom in vespids. Homologs of this gene or protein have been identified in many insect species^39^,^40^. However, the exact biological function and its sequence-related proteins remain unknown. Two ferritin proteins were also identified in *B. dorsalis* MAG secretions, which have previously been implicated in the immune response of ferritin in *B. dorsalis*^41^.

Interestingly, four pheromone/general odorant binding proteins (Obps) were identified in the MAGs secretions. They are traditionally associated with the olfactory nervous system^42^, and may present odorants, pheromones, or other small molecules to receptors in the female reproductive tract. Obps have also been identified in the MAGs of *D. melanogaster*^6^,^43^. Several Obps showed a MAG-specific expression in *D. melanogaster*^43^, but the specific functions of these tissue-specific Obps remain unknown. Two Obps (Obp21 and Obp2) were detected with high abundance in *B. dorsalis* MAGs by MS (Table 1). In *B. dorsalis* MAGs secretions, a further three Obps-like proteins (CRLBP homologous, AGAP011367-PA and GJ10540) were also identified by MS, and these are particularly attractive targets for further characterization. These Obps may be transferred to regulate the female reproductive physiology by interacting with a receptor in the female reproductive tract. An up-regulated expression of the Or10a odorant receptor has been observed in female *D. melanogaster* reproductive tracts in response to Acps^44^, suggesting Or10a as a possible target in *B. dorsalis*.

Two hormone-related Acps, which are similar to Obps, were identified in the MAGs secretions in the current study. One of them CG5867 was functionally predicted as a JHBP by Blasted in InterPro. JH has a profound effect in insects regulating embryogenesis, maintaining the status of larval development and stimulating reproductive maturation in the adult forms^45^. JH is transported from the sites of its synthesis to the target tissues by a hemolymph carrier, called JHBP. JHBP protects the JH molecules from hydrolysis by non-specific esterases. It has been demonstrated that JH could be *de novo* biosynthesized by the mosquito MAGs^46^, and that JH produced by the MAGs was transferred to the female ovaries during copulation^29^. A study in *T. castaneum* revealed that JH regulated the secreting activity of the MAGs in return^47^. Proteomic analysis in the current study revealed that one JHBP (CG5867) was the most abundant Acp in the MAGs. Results of qRT-PCR also validated the high and tissue-specific expression of CG5867 in the *B. dorsalis* MAGs at mRNA levels (Fig. 5). Previous study showed that JHBP was highly expressed in the larval immature stage, especially in the fat body^48^,^49^. Here in this study, we believe that the abundance of JHBP validates the presence of JH in the *B. dorsalis* seminal fluids, but the receptors for JH, and the mechanism of JH regulation of reproductive physiology, remain to be determined. In addition, we want to note that the crustacean neurohormone GA12379, which has neuropeptide hormone activity, was also identified in the current study (CL896). This protein belongs to the neuropeptide family that is expressed by arthropods^50^. The specific function of this protein should be determined in further studies.

In this study, we identified a number of Acps secreted by the MAGs of *B. dorsalis* using combined transcriptomic, proteomic and bioinformatics analyses. Most of the identified Acps in the *B. dorsalis* MAGs were functionally unknown proteins, but for 58 of these Acps we could functionally classify them into 11 categories, including protease, immunity, odorant binding as most important. For the new Acps in the male *B. dorsalis* MAGs, only 32 Acps would be functionally predicted by Blasting and searching in databases based on the sequence similarities and domains. We believe that the MAG-specific proteins as revealed by this study will be a foundation for future research to understand the patterns and processes of molecular evolution, mating regulation, and immunity among reproductive proteins in Tephritid insects. This LC-MS proteomic approach yields large numbers of proteins present in the secretions of the MAGs, and even less abundant Acps will be identified. Finally and interestingly, the most abundant of the newly identified Acps was identified as a JHBP which is confirming the impact of Acps with the reproductive event in the female adult. Future functional tests with the individual Acps or combinations will be essential for understanding their role in the female behavior and reproduction. Thus, our study provides important information combining proteome and mRNA data, and this for the first time, to address fundamental questions about reproduction and evolution within and among insect taxa, and also paves the way for further exploration of the functions of these Acps in the female adult. Finally and of interest to practice, this new information on insect-specific Acps may be useful in the development as new insecticide target sites, for instance to provoke male sterility and so to combat fruit flies that show high levels of resistance against all current insecticide classes.

## Methods

### Protein coding sequences prediction

The transcriptome sequencing was performed and analyzed in previous study with the accession number of SRR1168415 in the Sequence Read Archive in National Center for Biotechnology Information^20^. Unigenes were firstly aligned by blastx (*E*-value < 10^−5^) to protein databases in the priority order of National Center of Biotechnology Information NR, Swiss-Prot, Kyoto Encyclopedia Of Genes And Genomes (KEGG) and Clusters of Orthologous Group (COG). That is, we first aligned Unigenes to NR, then Swiss-prot, then KEGG, and finally COG. Unigenes aligned to a higher priority database will not be aligned to lower priority database. The alignments ended when all alignments are finished. Proteins with highest ranked in blast results were taken to decide the coding region sequences of Unigenes, then the coding region sequences are translated into amino sequences with the standard codon table. Therefore, both the nucleotide sequences (5′–3′) and amino sequences of the Unigene coding region named CDSs were acquired.

### Preparation of MAGs secretion samples

A stock colony of the oriental fruit fly *B. dorsalis* was established from pupae obtained from Haikou, Hainan Province, China, in 2008. The insects were reared in our laboratory according to methods described previously^51^. Adult male of *B. dorsalis* were dissected in saline solution (NaCl, 0.9%) at 15 days old after emergence to recover the MAGs, including two types of glands (mesodermal and ectodermal accessory glands) and the ejaculatory duct. Dissected MAGs were immediately immersed in 200 μL of sample storage solution (8 M urea, 2 M thiourea, 4% CHAPS, 1% (m/v) DTT, and 0.14% (w/v) PMSF) in a 1.5-mL tube. Fresh samples from 100–200 individuals were vortexed for ~20 s, and then centrifuged at 12,000 rpm for 15 min at 4 °C. The resulting supernatant was used for digestion. The protein concentrations were determined as described by Bradford using BSA as a standard^52^. Two independent MAGs secretion samples (biological replicates) were prepared. Protein digestion was performed as described by Dong *et al.*^53^. Briefly, secreted proteins were digested with trypsin (1 μg trypsin per 25 μg protein, Promega, Madison, WI) overnight at 37 °C in 150 μL of 50 mM NH_4_HCO_3_ according to the filter-aided sample preparation protocol. Tryptic peptides were recovered by centrifugation at 4 °C for 10 min in the ultrafiltration tubes, resuspended in 1% formic acid, and then lyophilized using Coolsafe 55–4 (Gene, Denmark).

### LC-MS analysis

Tryptic peptides were separated using an EASY nanoLC 1000 system (Thermo Fisher Scientific, San Jose, CA) with an EASYSpray column (C18, 2 μm, 100 Å, 50 μm × 50 cm). An acetonitrile gradient of 2–100% in 0.1% formic acid was used, and samples were run for 120 min at a flow rate of 250 nL/min. The separated peptides were analyzed using a Thermo Scientific Q Exactive mass spectrometer (Bremen, Germany) operating in data-dependent mode. Up to 10 of the most abundant isotope patterns with charge ≥2 from an initial survey scan were automatically selected for fragmentation by higher energy collisional dissociation with normalized collision energies of 27%. The maximum ion injection times for the survey scan and the MS/MS scans were 20 and 60 ms, respectively, and the ion target value for both scan modes was set to 1 × 10^6^. The spray voltage was 1.8 kV. Full scan mass accuracy was obtained by Orbitrap over a mass coverage of 400–15,000 m/z at a resolution of 30,000. Each sample was analyzed in triplicate.

### Data analysis and Acps identification

Proteins were identified using the MASCOT search engine (version 2.3, Matrix Science, London, UK). The raw data were converted to MASCOT generic files using the Proteome Discoverer software (version 1.4, Thermo Scientific). Mass spectra were searched against the transcriptomic coding sequences (CDSs) from *B. dorsalis* MAGs transcriptomic data (accession number SRR1168415 in the Sequence Read Archive (SRA) of NCBI). The initial precursor mass tolerance and fragment mass tolerance were set to 10 ppm and 0.02 Da, respectively. The search included variable modifications of methionine oxidation and N-terminal acetylation, and fixed modification of carbamidomethyl cysteine. Minimum and maximum peptide lengths were set to six and 144 amino acids, respectively. A maximum of two miscleavages was allowed in the data search. Both peptide and protein identifications were filtered at a 1% false discovery rate. In cases where identified peptides were shared between two proteins, the results were combined and reported as one protein group. MASCOT results were filtered using the MASCO percolator for accuracy and sensitivity, thus improving peptide identification^54^. A minimum of one unique peptide was required for protein identification. Proteins identified in both samples were considered to be the Acps candidates. Amino acid sequences of the ORFs of the selected Acps were then determined from the *B. dorsalis* MAGs transcriptome data. In this work, amino acid sequences with ORFs were screened out for the purposes of signal peptide prediction. Signal peptide prediction was carried out using the online tool SignalP 4.1 (http://www.cbs.dtu.dk/services/SignalP). ORFs of the proteins from the *B. dorsalis* MAGs containing a predicted signal peptide were considered to be Acps.

### Protein quantification

We used the intensity-based absolute quantification (iBAQ) algorithm to compare the abundance of different proteins within each MAGs secretion sample^55^. Label-free quantification was used to compare the relative quantification of proteins in each sample, with a minimum of two ratio counts to determine the protein intensity^56^,^57^. For this, we used both unique peptides and razor peptides to determine the label-free quantification. The mean protein intensity of proteins detected in both samples was estimated as the abundance.

### Functional analysis of Acps

Combined InterProScan (http://www.ebi.ac.uk/ InterProScan/) and BLAST similarity searches against the annotated proteins in the NCBI nr database were performed to determine the functional categories. The known Acps were analyzed using the online tool AmiGO 2 from the Gene Ontology Consortium (http://amigo.geneontology.org/amigo).

### RNA extraction for validation of novel Acps

Newly emerged males were dissected at 3 days old to obtain tissues from the head, thorax, midgut, fat body, Malpighian tubules, testis, and MAGs. Tissues were immediately immersed in RNA-later solution on ice and stored at –80°C until required. Frozen samples were powdered in liquid nitrogen, and mRNA was isolated using TRIzol reagent (Invitrogen, Carlsbad, CA) following the manufacturer’s instructions. RNA was quantified by measuring the absorbance at 260 nm using a NanoVue UV-Vis spectrophotometer (GE Healthcare Bio-Science, Uppsala, Sweden). The purity of all RNA samples was assessed at an absorbance ratio of OD_260/280_ and OD_260/230_, and the integrity of RNA was confirmed by 1% agarose gel electrophoresis.

The novel Acp candidates were assayed for tissue-specific expression patterns by quantitative real time PCR from standard concentrations of total RNA isolated from tissues. The primers in this study were designed using DNAMAN 7.0 (LynnonBiosoft, Quebec, Canada) based on the sequences of *B. dorsalis* (Table 3). For control purposes, a fragment of the ribosomal protein subunit 3 open reading frame was also amplified using the specific primers. Total RNA was reverse-transcribed using a PrimeScript RT-PCR kit (Takara, Dalian, China). gDNA eraser (Promega) was used to eliminate the genomic DNA. Each PCR reaction was performed within the StepOne Plus Real-Time PCR System (Life Technologies, Singapore) in a 20 μL volume containing 1 μL of cDNA template, 10 μL of GoTaq qPCR Master Mix (Promega), 1 μL of each primer (10 μM), and 7 μL of nuclease-free water. The reaction conditions were: one cycle at 95 °C for 2 min, followed by 40 cycles of 95 °C for 15 s and 60 °C for 30 s. Melting curve analysis from 60–95 °C was carried out for all reactions to ensure specificity and consistency of all generated products. Three technical replicates were performed for each trial. Transcript levels were quantified according to the 2^−ΔΔCt^ method^58^. Gene-specific primers were designed within predicted ORFs using Primer Premier 5.0 software (Premier Biosoft International, Palo Alto, CA). All primers used in the research presented here were available from the authors upon request. A control fragment of the ribosomal protein subunit 3 ORF was also amplified.

### Statistical analysis

Significant differences of expressions among tissues of male *B. dorsalis* for novel Acp genes were tested by ANOVA for multiple sample comparisons using SPSS 16.0 software (SPSS Inc., Chicago, IL). *P* < 0.05 was considered to be statistically significant.

## Additional Information

**How to cite this article**: Wei, D. *et al.* Proteome analysis of male accessory gland secretions in oriental fruit flies reveals juvenile hormone-binding protein, suggesting impact on female reproduction. *Sci. Rep.* **5**, 16845; doi: 10.1038/srep16845 (2015).

## Supplementary Material

Supplementary Information

## Figures and Tables

**Table 1:** Identification of proteins in the secretions of the MAGs of *B. dorsalis*.

No. of peptides	Annotation/Description	SC^a^	MW^b^	SL^c^	PEP^d^	iBAQ^e^
9	CG5867	62.9	29.091	264	0	111250000000
5	odorant binding protein 21	93.4	14.285	121	0	28666000000
4	heat shock protein cognate 3, isoform A	56.6	72.381	656	0	27481000000
2	protein disulfide isomerase	56.8	54.936	488	0	24224000000
12	antigen 5 precursor	49.8	27.727	251	0	20233000000
3	odorant binding protein 2	47.5	15.393	139	5.65E-42	14713000000
2	acidic ribosomal protein	58.9	11.469	112	2.79E-195	13052000000
5	GH20332	68.8	22.248	205	0	12351000000
15	Cyp1	65.7	24.764	230	7.12E-258	11974000000
14	ribosomal protein P2	52.2	11.743	113	1.09E-196	11773000000
3	GL15256	38.2	8.3386	76	2.87E-213	10433000000
4	GI22948	41.6	17.907	154	2.08E-185	8214700000
7	CRLBP homologous protein	54.4	15.957	147	0	7361600000
48	GI24315	47.8	91.335	797	0	6614700000
4	abnormal wing discs	47.4	19.035	171	0	5972900000
5	GI22236	53.9	15.073	141	2.59E-163	5311000000
7	GH13110	36.9	28.54	249	4.04E-119	4675000000
9	GH20247	60.1	20.616	183	0	4512500000
3	GI21205	40.8	37.646	338	0	3768200000
4	dromyosuppressin, isoform A	47.8	10.046	92	6.95E-155	3015000000
5	GL18529	36.1	15.582	147	1.30E-67	2551300000
9	GH18284	46.9	57.506	499	0	2530400000
16	GI21690	40.6	40.076	355	1.75E-127	1966500000
5	GJ18511	32.4	50.332	448	2.17E-188	1870900000
4	GA17249	12.2	36.103	335	1.39E-54	1792700000
12	GL17832	23.7	21.155	190	4.64E-97	1695300000
5	GF17355	30.6	54.810	480	1.42E-104	1678600000
8	GL19613	44.2	17.208	154	1.53E-162	1604600000
3	GM19093	46.4	24.506	211	0	1587800000
4	hexamerin LSP-2	28.3	83.016	699	0	1533600000
6	GI21056	22	17.710	168	9.36E-25	1511400000
4	GF14104	35.6	50.841	444	5.27E-105	1487000000
9	GF14758	36	29.037	261	3.83E-125	1374200000
7	ferritin heavy chain-like	28.9	23.143	204	1.50E-49	1370500000
10	CG9691, isoform A	44.8	12.540	116	1.49E-56	1157300000
14	GD13057	35.3	46.607	417	3.25E-241	1115500000
10	AGAP002632-PA	40	6.7478	60	1.63E-41	1035500000
6	GJ24273	32.5	28.130	240	1.87E-131	939140000
10	GF17838	37.9	22.684	206	1.90E-117	767750000
6	26,29kDa proteinase	26.9	62.407	547	1.76E-107	714250000
24	GG20623	37.9	50.194	451	5.81E-167	663700000
6	GJ10540	49.3	16.656	146	1.05E-26	657850000
4	GJ20558	26.1	37.215	330	8.64E-47	623240000
12	ferritin light-chain	26.9	25.119	223	7.48E-23	585380000
11	GJ12023	45.7	182.07	1630	0	579550000
6	GH20732	30.9	61.441	557	7.75E-116	544240000
5	GJ24273	21.3	31.237	267	2.51E-85	534720000
37	GK25863	30.1	142.76	1278	5.07E-242	523000000
6	GJ18745	18.2	31.872	291	2.43E-71	517470000
17	GD11983	29.6	39.172	338	1.21E-294	511620000
11	GG17054	29.5	26.828	241	1.75E-299	507980000
11	GH13810	30.6	7.8732	72	4.06E-11	496690000
4	GI22092	27.6	25.186	221	4.71E-216	482670000
12	GE26334	35.5	25.634	234	1.94E-45	479020000
2	Chitinase-like protein Idgf4	40.7	47.835	435	1.22E-72	445130000
6	GF23312	49	17.015	153	3.05E-27	424600000
6	GJ19442	26.6	32.776	297	8.07E-93	418670000
9	GK12751	27.6	46.727	392	8.23E-80	368820000
3	GL12691	47.4	8.4289	76	1.24E-16	354380000
11	GI15820	32.2	16.243	146	6.87E-13	343610000
12	odorant binding protein 1	43.5	15.890	138	1.58E-20	339060000
8	GF24261	36.5	27.134	244	2.45E-74	332140000
4	AGAP011367-PA	48.5	26.308	229	1.75E-42	300750000
5	GF24406	24.1	62.360	551	1.37E-100	295230000
23	GF13366	35.4	81.191	726	4.67E-113	284410000
5	GK24231	22.9	15.056	140	5.62E-13	258140000
2	GF23809	33.3	8.997	81	1.05E-08	257940000
7	serine protease inhibitor 1	25.6	44.468	406	2.17E-37	254430000
2	GH21921	23.5	47.212	412	2.28E-51	248570000
5	GA12379	45.9	14.332	122	1.48E-15	238840000
5	Gmfb8	35.8	24.959	218	2.01E-28	226580000
3	GA15794	15.4	43.383	396	3.99E-57	208850000
3	gasp, isoform A	49.6	28.454	258	5.83E-140	199290000
4	obstructor-E, isoform A	36.5	9.0422	85	4.95E-06	195140000
2	CG31195	21.3	93.729	802	1.44E-84	173140000
4	GA19485	29.5	23.525	210	1.05E-15	155040000
10	GH20023	27.4	20.419	175	7.72E-15	149910000
16	cellular repressor of E1A-stimulated genes, isoform A	22.1	21.481	195	2.70E-15	142470000
5	juvenile hormone epoxide hydrolase 2	14.9	52.745	464	7.34E-26	126460000
7	GA20648	14.2	39.852	331	1.21E-12	115880000
3	maltase A5, isoform A	25.5	10.375	94	3.66E-08	111750000
25	insect-derived growth factor	22.3	61.333	539	1.65E-37	86078000
6	cathepsin F like protease	13.1	52.459	467	2.45E-14	82380000
12	GK15467	22.2	11.754	108	3.54E-07	77075000
6	putative zinc-metalloproteinase precursor	15.7	28.380	249	1.13E-14	75032000
14	Gp150	15.1	102.620	911	2.96E-45	57972000
5	GJ17158	12.1	28.285	256	1.30E-13	56852000
9	GJ23716	19.2	61.573	546	2.52E-111	49834000
2	putative salivary trypsin	15.4	44.870	408	1.62E-32	23734000
5	GJ18156	8.8	71.421	617	1.29E-13	17199000

^a^Sequence coverage (%).

^b^Molecular weight (kDa).

^c^Sequence length.

^d^Posterior error probability of the identification.

^e^Intensity-based absolute quantification.

**Table 2:** Function classification of proteins in the secretions of the MAGs from *B. dorsalis*.

Functional class	Number of proteins
Protease	10
Protein binding	13
Ion binding	6
Immunity	8
Odorant binding	5
Hydrolase	4
Protease inhibitor	4
Structural protein	2
Chitin binding	2
Hormone binding	2
Protein modification	2
None predicted	32
Total	90

**Table 3:** The primers used in the tissue-specific expression profiling study for the six most abundant and novel Acps in the secretions of the MAGs from *B. dorsalis*.

Gene	Forward Primer (5′–3′)	Reverse Primer (5′–3′)
*RPS3*	TAAGTTGACCGGAGGTTTGG	TGGATCACCAGAGTGGATCA
*CG5867*	CTGGACATCGAGGCAGAAGG	CCAAGTTGTGCGCGTTTCTT
*GH20332*	GGATTATGTCTGGTGGCGCT	AAATTGCGGGCGGTTTTCTC
*GL15256*	CTGCTGGCCGTATTGCTACT	CGCAGCATATTCAGACTGCG
*GI22948*	TATCTGGGCGGTCTGGAAGA	CGCAGCTTTCATCCCAATCG
*GI24315*	GCGGTTCCAATCATCCAACG	ACCATCGAACTCAGGCAAGG
*GI22236*	TTGCGATCAGGTGCCAAAAC	GGGAGACGGTGCAAAGATCA
