# Supplementary material for: Proteome analysis of male accessory gland secretions in oriental fruit flies reveals juvenile hormone-binding protein, suggesting impact on female reproduction
Source: Sci Rep. 2015 Nov 19;5:16845. doi: 10.1038/srep16845 (PMC4652233; doi:10.1038/srep16845)
Supplement: Supplementary Information [file srep16845-s1.pdf]

Supplementary Section

Proteome analysis of male accessory gland secretions in oriental fruit flies reveals juvenile hormone-binding protein, suggesting impact on female reproduction

Dong Wei[1], Hui-Min Li[1], Chuan-Bei Tian[1], Guy Smagghe[1,2], Fu-Xian Jia[1], Hong-Bo Jiang[1], Wei Dou[1]& Jin-Jun Wang*

[1]Key Laboratory of Entomology and Pest Control Engineering, College of Plant Protection, Southwest University, Chongqing 400716, China.

[2]Department of Crop Protection, Ghent University, B-9000 Ghent, Belgium

Correspondence: Dr. Jin-Jun Wang, College of Plant Protection, Southwest University, Chongqing 400715, P. R. China. Tel: (86)-23-68250255; Fax: (86)-23-68251269. E-mail: wangjinjun@swu.edu.cn or jjwang7008@yahoo.com

>CG5867
MSRISYVLVILACLNGWCAASFQLKVDMPKLPNITPLTALPPGIPTCVGTNSDLNACIKNGLKEIKPRLKSGIPELSI
PPLDPLVLGSHKVEMTDDFAKGYLNVHNLIIKGISESTVDTLNLEMQGDHVKLQVTTKTPSIEKQGAFQGELTAEG
LNLKPEGQFTSKLTDLQLDIEAEGDLTEREGHKYLQLKSFNLIPQIGDLEFNADNIVPDKGLNTVILAVINSHWPTF
YKLLVKETRTTWEPIVLFVANAYLTAVPFDLFI

> odorant binding protein 21
MKYFMFIVILAVVALVQADDWSPKTVDDIKKIREECMKQVPSSDEEFQKRKENDYPDVESVRKYALCNSKGWGL
YKEGKGFYPDRVAEQFKDDMPEDEIKAIVNDCDEKTKEETDDERCYHLLKCVMSTKLGDHIKDLVKRLE

>heat shock protein cognate 3
MKVCLLLLAVVAFVSLSHGEEKKEKDKDIGTVIGIDLGTTYSCVGVYKNGRVEIIANDQGNRITPSYVAFTADGER
LIGDAAKNQLTTNPENTVFDAKRLIGREWSDSNVQHDVKFFPFKVVEKNSKPHISVSTAQGNKVFAPEEISAMVL
GKMKETAEAYLGKKVTHAVVTVPAYFNDAQRQATKDAGVIAGLNVMRIINEPTAAAIAYGLDKKEGEKNVLVFD
LGGGTFDVSLLTIDNGVFEVIATNGDTHLGGEDFDQRVMDHFIKLYKKKKGKDIRKDNRAVQKLRREVEKAKRA
LSGSHQVRIEIESFFEGEDFSETLTRAKFEELNMDLFRSTLKPVTKVLEDADMNKKDVHEIVLVGGSTRIPKVQQL
VKDFFSGKEPSRGINPDEAVAYGAAVQAGVLSGEQDTDAIVLLDVNPLTMGIETVGGVMTKLIPRNTVIPTKKSQIF
STASDNQHTVTIQVYEGERPMTKDNHLLGKFDLTGIPPAPRGIPQIEVSFEIDANGILQVSAEDKGTGNKEKIVITN
DQNRLTPEDIDRMIRDAEKFADDDKKLKEKVETRNELESYAYSLKNQIGDKDKLGGKLSDDEKTKMESAID
DTIKWMEQNADADPEEYKKQKKDLEAIVQPIIAKLYQGAGGAPPTEESDDDAKDEL

> protein disulfide isomerase
MKFVISSLLLVAVIASGAYAAEEVKLEDGVLVLTTDNFDEVIKNNEFVLVEFYAPWCGHCKALAPEYAKAAQALA
EKESPIKLGKVDATVEGNLAEKFQVRGYPTLKFFRNGVPVEYSGGRQSADIISWVNKKTGPPAKELKTVEEAEKF
LKDNEIAVVGFFKSQESDEAKAFISVANALDTFVFGLITEDELISHYEAKDGAVVLFKPFDEKKAIYEGEHTVENIK
KFVQVQSLPLIVEFNHESASKIFGGSIKSHLLFFVSKEAGHIEKHVDPLKDIAKDYREDILFVTISSDEEEHQRIFEFF
GMSKEEVPTIRLIRLEEDMAKYKPESNDLSASTIKEFLQKFMDGKLKQHLLSQEVPEDWDKNPVKVLVASNFDDV
ALDKSKDVLVEFYAPWCGHCKQLAPIYDQLGEKFKDNENIVVAKIDATANELEHTKISSFPTIKLYRKGDNKVIDY
NLDRTLDEFVKFLEAGGDVAQSGESEEEVEE

> antigen 5 precursor
MEPKFLLLASFIVALQIVCAQDYCDSSYCGSRKHIACNNNGAFAASCQSPAMVTFTQAEKNSIVDAHNAKRNTVA
GGKTALKPACRMATMQWDDELAKLAAFNVKQCQMKHDSCRNTKTFKHSGQNLARRSFNSSPNITQLSLLSVDA
WYNEIKDTKMEYMNAYPSSYKGPAIGHFTVMVADRNIRVGCAASTYRVSGHRYTAFLFACNYATTNMVNFPIYKS
CSVAASQCTTGKNPSYTSLCSASEKYDVNKWF

> odorant binding protein 2
MKFCLALLSLLMVVVFAVADHAGHTDYVVKTNEDLIRYRDECVSKLSIPSDLVDKYKEWSFPDDEKTRCYLKCV
LEKFELFDAAKGFDVHNIHHQLVGANADHSDATHGAIENCAKEAAGDDACVRAYNGFTCFLKNNAQLVQAGVE
KSSK
> acidic ribosomal protein
MSTKAELACVYAALVLVDDDIAVTGEKISTILKAANVEVEPYWPGLFAKALEGINVKDLITNIGSGVGAAAPAGG
AAAAGGDAAPAEAKKEEKKKEEEPEESDDDMGFGLFD

> GH20332
MKLYLGLCLVALAAGIVVADESKGPKVTDKVFFDITIGGEPAGRIEIGLFGKTVEKTARNFKELAEKPEGEGYKGS
KFHRVIKDFMIQGGDFTKGDGTGGRSIYGERFPDENFKLKHYGAGWLSMANAGKDTNGSQFFITTKPTSWLDGR
HVVFGKVLSGMSVVRKIESYETDGRDRPVKDVVIANSGSIPVDEPFSVAKTDATD

> Cyp1
MALFGATATLLRGCCRQQRPLAILAKKSNFLQTGIEVGQLHFEFQSQIGFLGVRFASNSSKSNKGKMGLPRVFFDM
TADGQPLGRIVMELRPDVVPKTAENFRALCTGEKGFGYKGSCFHRVIPNFMCQGGDFTNHNGTGGKSIYGAKFA
DENFSLKHTEPGILSMANAGPNTNGSQFFICTVKTAWLDNKHVVFGKVVEGMDVVKAIEGLGSQSGKTSKKIVV
AESGAL

> ribosomal protein P2
MRYVAAYLLAVLGGSENPKNADIEKILSSVGIEVDSERLTKVVKELNGKSVEELIAQGREKLSSMPAGGAAAAAA
APAGGAADAGGDKKEAKKEEKKEESESEDDDMGFGLFE

>GL15256
MQFLRFITSLLLAVLLLATLTTAAPAGENDGPFCPCPRNYDPVCGSNMVTYSNRCNYDCVRRDAERRGRSLNMLR
SGPC

> GI22948
MRSLLLVMAFAFVLIHSSIALKEDECEVCVKTVKRFADTLNDDIKKDHKKIEAEFKSFCKKQKNKEHRFCYYLGG
LEESATGILNELSKPLSWSMPADKICEKLKKKDAQICDLRYEKQIDLNNVDLKKLKVRDLKKILNDWDESCEGCIE
KTDFIKRIEELKPKYAHNEL

> CRLBP homologous protein
MAKFILFAALCILSAAVSNAAFNKEEAIKNFMTRAEECRGEVGAADSDIQDIVAKVPASSKEGKCLRSCLMKKYG
AMDSNGKFVKSVADQHAQDFTDGDADKLKTAREIIDACADIAVPDDHCEATEVYGKCFMDQAKAHGMQKFDF

>GI24315
MKYLLLLALLLLAGVGEIRADESPEDVETIDLDLGSFKEGSRTDAETLKREEEAIKLDGLSVAQMKEVREKAEKFT
FQTEVNRMMKLIINSLYRNKEIFLRELISNASDAIDKIRLLALTNKGELDTNPELNIRIKADKENKILHIMDSGIGMT
HQDLINNLGTIAKSGTADFLSKMQDTSKTESGQDLNDMIGQFGVGFYSAFLVADRVVVTTKHNADKQYIWESDA
NSFSIVEDPRGDTLKRGSIISLHLKDEAQDFLEEDTVRELIRKYSQFINFPILMWSSKTIEEDVPIEEDSTTEESEKAE
DNLEDSDDEAKVEEESENVDKPKTKKVSKTVWDWQLINDSKPIWTRNPADVTDDEYTEFYKTLTKDSSEPLTHT
HFVAEGEVTFKSLLYIPKVQPSESFNRYGTKADNIKLYVRRVFITDEFNDMMPNYLNFIRGVVDSDDLPLNVSRET
LQQHKLIKVIKKKLVRKVLDMIKKIDKDNYSKFWKEYSTNIKLGIMEDPSNRSRLAKLLRFQSSNGKDLTSLAEY
VERMKAKQEHIFFIAGANRAEVEKSPFVERLLSKGYEVLYLVEAVDEYCISALPEFDGKKFQNVAKEGFKLNESEK
SKSRFEELKSTFEPLVKWLNEVALKDKILKAQVSERLSNSPCALVAGMFGWTGNMERLALSNAHQKSDDPQRSY
YLNQKKTLEINPRHPLIRELLRRVEADEADSTAKDMAVMMFRTATLRSGFMLQETSDFADTIEKMMRQSLGVPLD
EQIELDEDDEDIAGDTGNDEENDNLSENKDSEDDEVHDEL

> abnormal wing discs
MSSRSFNRSFSLINLLGKKAVMIGTILAFFSLFSYAMAANKERTFIMVKPDGVQRGLVGKIIERFEQKGFKLVAMKF
TWASKDLLEKHYADLSARPFFPGLVNYMSSGPVVPMVWEGLNVVKTGRQMLGATNPADSLPGTIRGDFCIQVGR

NIIHGSDAVESANKEIALWFNEKELVSWQPAAEGWVYE

>GI22236
MKQIIALVLFAACLLPSGDAIKCYQCKSLTVANCAKDVIDDSLNIPSVDCDQVPKPNTMDQLLPVTKCNKVVTSD
KAGIIVSRDCHFEVVGQKPDLCTVSHSREVQSCHICKGDLCNASSAGFVAISLAALISLIAMQFLM

>GH13110
MRTLLVLALLAMISGSHTLPTVIQWEEDPEQTPGFFEGDIVLRPKSRNCMSNPSQHWPDGIVYYKFSEGIDDVRKD
FIRNAMNIVEEGSCVRFKEADDDQPYFVNITGNPGGCYSTVGFVEDISILNLHKHDLNTGCYRTGKIIHELLHTLGF
YHMQSTYDRDDYIRIAYENVKPDFVHDFAKYSKDFVEDFGQKYDYGSIMHYSPHAFSANGAETIVPLQEIREGLM
GQREALSTVDLIKLNKMYKCP

>GH20247
MIAKALIKVVCLLVLWSVAEGFTSQEVKCHVCKATVQELENAISKEDPNKTVDVSGFRLDARGNSISKSVKLVKS
EMYLTELMEKICDKMEDYVKATYKSNGKFTLLKMIVDGKMNPESSLVDFVQDGDLNKSLGHYCLEILDDHEEIII
KALQAPTLSDELDTQICGAQAKYCPYSPIQEEYDFDEKDEL

>GI21205
MRAVLIFVALFAYASAVSFSDLVKEEWFAYKMEHKKHYSNEVEERFRLKIFNENKLKIAKHNQLYAAGKVSFKMA
VNKYSDMLHNEFRETMNGYNNTLRKQLRSNRKFTGATFIAPAHVTVPTSVDWRSHGAVTDIKDQGHCGSCWAF
STTGALEGQHFRKTNTLVSLSEQNLVDCSGKYGNNGCNGGLMDNAFRYIKDNGGIDTEKSYPYEGIDDSCHYNPS
SIGATDRGFVDIPAGDEEKMKQAVATIGPVSVAIDASHESFQFYSEGVYNEPACDAEQLDHGVLVVGYGTDPSGQD
YWLVKNSWGTTWGDKGYIKMARNKDNQCGIASASSYPLV

>dromyosuppressin
MSSQMFVFVCFATLLIAAAQSSNAMAVPPFCQSGMVEDMPPHIQKVCSALNNSEQLATALKSYLNSEAAALLTDQ
APLKRSDVDHVFLRFGKRR

>GL18529
MVSAVKSLLAVAVIASVACSAFAVQCYICDSVTNPKCGQKFEASDDMKYDCARVSPPRFLQNFFNVHNATGCMRK
VLDIPGHPQVIRGCFYGDVSNTQSGCQADPSLPFVKQLSCDVCSGNLCNGSSATAPIAAAIVLFFALARMLS

>GH18284
MVVPLNDMLPFATGERKLAACVLLLLLLELFLEGADAVPNQADIENHLELGKEFLARGQLADALTHYHAAVEGDP
NNYLTLFKRGTVYLALGKARFAIQDFTRVLELKPDFAAARIQRGIVHLKSGEYEQASEDFEHVLQEDPYNESVNE
HFRRIEAAIEQWEIVKDLRYRGDSRNIIPMITQLLEISPWSIEFRQARADAYIAIDELPSAISDLRSVNKLSQDSTEGY
YNIALLLYKMGQATNALKEIRECLHLDPEHKDCFPFYKKLRKVEKSLANAESSREEKQYADCINSAESVLKQEKN
EKMVIYEAKRLLCTCYTRDEQFAKALSYCKEALDVLKDPQLYCERADAYIGSEMYDDAVHDYQRALEMDESLQ
RAKEGIEKAKRLQKQAERRDYYKILGVKRTANKQEIIKAYRKAAQKWHPDNFKDDEKKIAEKKFIDIAAAKEVLT
DPEKRKQFDMGQDPLDPEANQQQGGFRGGSPFAHFHHGSPFQFKFHFN

>GI21690
MRTLAFILLVQIAFCLLSVAFAGRDFYKILNVKKSASTNEIKKAYRKLAKELHPDKNKDDPSASEKFQDLGAAYEV
LSDPDKRKTYDRCGEECLKKDGMMDHGADPFASFFGDFGFHFGNGDPHEHQTARGANIVMNLYVTLEELYSGN

FVEIVRNKPVLKPASGTRKCNCRQEMVTRNLGPGRFQMIQQTVCDECPNVKLVNEERTLEVEVEAGMVDGQETR
FVAEGEPHLDGEPGDLIIKIMQTPHKSFQRKGDDLYTNVTISLQDALIGFTMNITHLDGHAVSITREKITWPGARIRK
KGEGMPNYENNNLQGNLYITFDVEFPKKELTDAEKEDLKKILDQASINRVYNGL

> GJ18511
MWKYFVFICVFLQTSNAVLESDANTLVLLDNLAIRETHSIFFKSLQERGFKLVYKLADDSGLVLSRYGEYLYKNVII
FAPSVEEFGGDLSVEKLTEFVDDGGNVLVAASEQSGDALREFASECGFEVDEEGASVIDHLNYDVSDVGDHTTILT
SARNLIDAPIIVGAKKSAPLIYRGTGLLADKENPLVLHLLSAESTAYSYNPEQTVKEYPHAVGRETLLIAALQARNN
ARVVFSGSLHFFSDEAFTAPVQNARDGTINKQASNQDVATAISRWAFGESGRLRVAKVNHHKEGESLPPEQAYTIV
DPVVYTISIEELVEEKWQPFKANDVQLEFVRIDPFIRTTLKQVNGGRYEARFKIPDVYGVYQFKINYDRVGYTHLY
NTTQVSVRPLEHTEYERFIPSAYPYYSSAFSMMIGVFIFSFVFLHFKDENNTVSAKSGKADDKKMQ

>GA17249
MFHNKYLTLTLVLSICSALVCAAQIDVSLNNAKLPADAASAAPVVAKDESVEKPKSTTPTTTSSTSSTTTTTSTTTT
STTTTTTPAPTTAAPTTTTVAPTTPAAPNTTTVAPITTTPAPAPFPAPEIGMWNSSCIIMHFAAQLNVTYETKDNKIAS
RLYNIPKDAKVEDSNCANISQTIHIIWGPVEAVHSMVLQFDMVNKTSELKQIYITLPLTSEHFPDAKDNETIQLIHN
GDEFVTPVQMSYHCTRAQKFNMTEVMQDNKVIGTITLSNVQTEAFITDHRNTFSTAMDCDGPKTMDIVPIAVGIA
MAALILIVLISYLCARRRSTSRGYMSF

>GL17832
MFKSLFSVVILLALSNICLSDDETSARLLISKQILNKYLVEKSDLLVRYTIYNVGNGAATNVKLVDNGFPSEAFDVV
GGQPTATIERIAPQANYTHVLVVRPKAYGYFNFTAAEISYKPVEEAEELQLAVSSEPGQGGIINLAEFNKRFSSHFFD
WVAFAIMTLPSLAIPLALWHSSKSKYERYGKNKKH

>GF17355
MKNAIHVALFGLMALSFFKEGNAKKLKFARSFINPYPRFQEYSDGGDPGEPLYLTPLIHDPKIPNDQVRQKAAVVG
SQFHKVESYSGYLTVDAARKSNMFFWYFPAEEYPDYAPVVLWLQGGPGASSLFGLFVENGPFEFDSHGHLQKRN
YTWSRSHNLIYIDNPVGTGFSFTGSDDGYARNEKDVGRNLHEAVMQLYELFEWSNSTGFWITGESYAGKYVPALA
YHIHLMQNAVDTRVYIPLKGLAIGNGLSDPLHQLKYGDYLYQLGLIDDHGLNRFHIEEEKGKKCIEQHDMDCAFE
VFDGLINGDLTNGSVFHNLTGYNYYYNYLKTDSDLPNDVLGNFIQSSATRRAIHVGNKTFHDLDKENKVEKFLKK
DVMDTVAPWIAELLKDYIVCIYSGQLDIIVAYPLTRNYLMHLKFADADKYKIADRKVWQVDGEIAGYAKHAGHLI
EILVRNAGHMAPGDQPKWLEWMIHHLTHYK

>GL19613
MASTTSAKALFISTAVIMLTFCCFTPTVNAIRCHQCNSHLQEDCTELRLITPRAPRDEQFLTECESPDMFCRKTITKI
EVSGENRIIRSCGYLDNDKSAKTNYCFDADNEGYKQRICTCYEDGCNAAPPRLGNANHVTMLSATGLCVLVARF
LR

>GM19093
MALLTMIARVIDGLPLVGTMQDDEQSGRSILEYQNQAKMLFRKLGSHSPARCSIETGPYLFHYLIENDVCYLVMC
DKMYSKRLAFNYLEDLAQEFHANYGRRVNSVTRPYAFIEFDVYIQKAKKQLTDRRRNINAINTQLQDVQRIMVQ
NIDDVLQRGTVLSELDTKTQNLSMMSQKYKKDATYLNRKSMYLKLGAAGVAIVVFILYFWVL

> hexamerin LSP-2

MKSYTLVAFAALALCATVSTKNIESKTADKDFLIKQKFILEILQHVYQDDVLVTKYDTSYYEYKPWEHVADYHKH
ELLEPFFELWQHKPMHDDEVFSIMYERHVEYAVGLTRLFYFAKDWTTFTHAVFWARLHVNKQLFVYALTVAGLH
RADMQGIVYPAIYEIQPWYFFDVETIETAERYRMHNFHNVKKLDNIYNVAIKSNYSNVYSNMHRDHELAYFLEDV
GLNAFYYYYNLDYPFWTKGVEGMELNKDRRGEFWIYTHWQLLARYYLERLSHDLGDIEEFDMYESIPNGYYSG
LRYYSGVNFPNRENGYSFYHNYNLEYIRLVNLVSQRIMDYIHDQHKDDIEAVNKLGNILQGNVDSIDRVRYSSLSN
YYKQIVNDGNDYGKYEETLPNTFMHYETALRDPLNFQIIKDIIHFYWHLVEVFPEYTVKDYNFEGVKINKVQMPD
HLTTYFEYFDSDISNAVNVEIPAEASADPLVNFGRNSQQDGQSFVVKARQYRLNHKPFQFQLDVTSDKAQKAIVK
VYIGPGQEEDKYHFIESNYMNFFELEHFVVDLVEGVNVITRNSDDFSWWVEDRTPYLELYKKVMDATNSDYKFG
LNQKEAHCGVPQRLMLPIGKKGGMPYQMFFMVYPYHEPAVKQHTGYDPIISCGIGSGARWVDSLPFGFPFNRPV
KHGYYFDVDNFHFEPVVIYHKEDAANVV

>GI21056
MSRNLLAVFLVCLCTGAALATTCTSPKVSVTSFSTQDATILTQVAHVGEFSLSCGNNAQPNLFAEFPCGKIVPVAKI
GDGKYQVSWTQEIKKSGGGNVAVRLFDEEGYANVRKAQRDGDKVANVKSLVDITVATKSAYKGPWVQAELVAA
LAVGGIAYFAFTAKSKVQ

>GF14104
MKTNRCSILLILIFYTICLTQAKIINKNVDRSLDISTQLVKELTKITATNSDGKPISVYTFIVPAADRKNLAFIAARDEN
KKELKYTEKVVDGNHQFSIAFPKSTATEVFTIEAVYTKKLIPHPSQIPQSGKQLVRYEGNLYLNSLYETKSQKLSVL
LSSANILSYTQTKPFNVASNKIKYGPYENIEALSNQSLVIHYENERPFLTTTRLERIIEVSHWGNIAVKENLYMEHTG
ALLKGSFSRYEFQKDARSGHAIKSYKTILPASASDVYYRDTNGNISTSNMKVMRDFVELELRPRFPLFGGWKTHY
TLGYNIPSFEYLFNAGDNYQLKMRLIDHIYNDMVIDEAEIKIILPEGVSNIEITTPYPVKRLPNEVHYTYLDTVGRP
VIIMSSKNLVENHIANFNLKYSFSKITLLQEPLLVVAFFFIIFIIAIISIRLDFSISTTHSHKD

>GF14758
MEAKFLASVISFLSIFLAIYADTEFLVPNYDNAEHQLKVYEIRSPLVLSCNITKAGDYVLKWEKNGTDVAKVKELA
GRYRIIAAERKFIIDRTDVADDGLYSCVADDQKKEINVVARVVVRVPSNTGVVEGEKLSIVCTSVGTDPQLSWMIG
NLTISNSTGRYILKPDDNHVKNAILSVENISLDDRGEYKCIGRNDANEYAGYADASDASFVRVKGKLAALWPFLGI
CAEVLILCAIILIYEKRRNKSELEESDTDPQDQ

> ferritin heavy chain-like
MMKLLAAVLLLTVAVNAQMTCRLEQPRIPVEWIGLNDKSGQCLEEMRKQIQMEINASNIYLAMAAHFSRDVVNR
PGFAEHFFKSAREERQHGSKLIEYLSMRGQLTDSVTDLIQLIDVDVKVDSGVDALRQALELETKVTKSIRSLIKVC
EKTPNWYHLVDWLTGEFLEEQLTGQRDLAGKLSTLTKMMATQGALGEFLFDKQL

>GD13057
WRKTRKPVTKFCAGTDGNRNFDFHWGEVGASNSACADTFRGPTAFSEPEMQALRDLMHSLTGRAKFYLSLHSY
GNYLLYPWGYTSALPENWHDIDDIALAGAAAIKTATGTEYTVGSSTNVLYAAAGGSDDYALGKARIPISITMELPS
AGQQFDPPLSKIEELASETWIGIKAMAEKVIEKY

>AGAP002632-PA
MKVLFFLALFVAAICAVMGQSCPTNFKWDSGNKLCTAERPIHGECPPNSQYNLNYNKCVY

>GJ24273

MRPEIKSFHRKQLPAFSLMLLSLMLFGFTSAQRVAPGVPPQHYQQVPVQHNQQPQYQQQQQQIHHAPNVNPQH
YQQQVQYQQVPVQQQQQQQQYQQQQQPQFQQQQPPQQQQQFQQQPPQPVQQQAPHAPHQAHHGQQQVLNA
GNIQDERAHIQEHMQVPIDTSKMSEAELQFHYFKMHDSDNNNKLDGCELIKSLIHWHVQGAHHKADEHHGTEE
EEKGKIYSDEQLTDIVDYVLKQMDLNNDGYVDYAEYRKSEDAQSSDDKKKP

>GF17838
MLKTEIKWSVLLLLQFVFVVLAGAQGGQSVAADKLDVTILYESLCPDSIRFMGRQLAPAYGNLKQNLNVNLVPFG
KSRSVNHGSEFYCQHGPAECAGNRLQSCVLNQPSTQDQRVRFAICQMLANDKQNVEECTDLVGLSSDVDGCVKT
NVGTQLQLLAEQVTNQYSPSFVPTIIYDGVFNQQLQDASQYDFRGTVCALLQKRGIV

> 26,29kDa proteinase
MHFAVFLLLALCGFSAEVLATKPPKWDETYITKGTLYIPYAEIAEPFYAWYDKTTKRSRIDYYGGMVKTYQLASV
MPYGTSLKLAPITTDEELNKVTCLQLNGTAENRVQIQTILPDARNFTLLGTESFLGFTCDKFRLEEVIGEKRNVYTL
WVRYKKSPHYPAARQPIPVRYEMRGYNTLLGSHFDHYFLDYDSYEHDDIPNEVFELDDNMPCVAFPGPGAGHYA
TFNPMQEFVHPAKDEHVEHSFNHFKAKHGIKYKTDKEHEYRKNIFRQNLRFIHSKNRARLSYTLAANHLADKTE
EELRARRGFKSSGIYNTGKPFPYNIEKLKDDLPDQYDWRLYGAVTPVKDQSVCGSCWSFGTIGHIEGAYFLKNGG
NLVRLSQQALIDCSWQYGNNGCDGGEDFRAYQWMMKMGGVPTEEDYGPYMGQDGYCHVQNLTLVAPITGFVN
VTSGDSNAFKIALLKHGPLSVAIDASPRSFSFYAHGVYYEPECKNGLDELDHAVLAVGYGTIQGEDYWLVKNSWS
TYWGNDGYILMSARKNNCGVMTMPTYVEM

> GG20623
MKILLVFSLFIAATLANKVGSPVPAGPKKHMVCYYDSSSFVKEGLGKLIIDDLEPALQFCDYLVYGWAGIERDSHK
AVSLNQNLDLDLGKGLYRTVTRLKRKYPTLKVLLGLGGDKDIETSEDAKDLPNKYLELLENPAGRARFINTVYAL
VKTYGFDGLDVAWQFPKNKPKKVHSGIGSLWKGFKKVFSGDHIVDEKAEEHKEQFTALLRDVKNEFRPDSLLLS
TTVLPNVNSSLFYDVPAIVNYLDFVNLGAFDFYTPERNPEVADLPAPLFDLPERNPQFNVHYQAQYWLRNNCPAS
KLNVGVPTYGRVWKLTDDSGETGVPPVAKVENEAPAGPNTQIKGLYSWPEVCALLPNTNNAYGKGANLALTKV
GDPTRRTGNYAYRSDEKSSGHGLWVGYEDPDTAAEKAGYVRQFNLGGVALFDLSFDDFRGSCTGDKYPILRAIK
YRLVN

>GJ10540
MKFFIVILAVVALAYADEEWVPKNVAQIKAIRQECIKDFPLSEEYIQKMKNFEYPDEEPVRKYLLCTAKKLGVFCE
HEGYHADRVAKQFKMDLDEAEVIAIAEGCADKNVEGSSADVWAYRGHKCVMASKIGERVKAYIQKSVEEA

>GJ20558
MNVLRRTVLLGLLVLAGFLVFTAAQGKQKTGLSLSEKVRNLHDMNMKKALLRFNGQKFREYVKNAPRNYSVV
VMLTALAPSRQCQICRHAHDEFTIVANSYRYSPTYSNKLFFAMVDFDEGSDVFQTLRINTAPVFMHFPAKGKPKGP
DTMDIHRVGFAAEAIAKFVHERTDVQIRIFRPPNYSGTVAMITLVALVGGFLYIRRNNLEFLYNKQIWGAVALFFCF
AMISGQMWNHIRGPPLVHKSKNGGVAYIHGSSQGQLVVETYIIMFLNAMIVLGMVLLTESGTQSDQKRGRIMAIA
GLLLVVVFFSFLLSVFRSKAQGYPYSFLFK

>ferritin light-chain
MKPFTALTLFAGIIALASADNYACHSNVTVGCSNSASTPVVCHSRYGGIENVESTIQSYINLNLHKSYQYLLLSSFY
NSYQKNRPGFNKLYRDLSDRSFDDAIDLIKHVTRRGGQVDFRAISQHPNSIAQSRLKLEEDELHSLGIALDIEKTLT
ADAGHVHYQSTHGRPHDPQTAHYIEEKFLHKQADSVRKVSGFVNDLSKIMNQPDPSLGIYLFDQYLEKQ

> GJ12023
MRLNKWSFFALRRQLLCGTLLLCLFMQHAPTTTAVGHGNTFMPALECYDQYGRPQRCMPEFINAAYQLQIEATN
TCGENGDNHFCIQTMNPYHKNCEICRYEDHHPAFLTDLHDPQNPTWWQSETMYEGIQHPNHVNLTLHLGKSYDI
TYVRILFRSPRPESFTIYKRTSESSEWIPYQYYSADCREQYSMTDSRAILKGEVEAHALCTSEYSDISPLRDGEIAFS
TLEGRPSGIIFERSSELQEWVTATDIRITLDRLNTFGDELFYDPQVLKSYFYAISDIAVGARCKCNGHASKCVTSTG
MHGERTLVCECRHNTDGPDCEKCLPLYNDVKWKRATSTEVNECKACNCNGFADKCFFDAHLFNLTGHGGHCLD
CRENRDGPNCERCKENFYMREDGYCINCNCDPVGSRSLQCNSHGKCQCKPGVTGEKCDRCDNNYYQFGQHGC
QHCGCDPRGSADNVPSCNPNSGICHCKENVEGKRCNECKPGFFNLDQTNRFGCTPCFCYGHTSECQTAPGYSVVS
VTSNFNKHKERWTAIDLYNRDVDIKYNQYSRSIGTTAQGNEYIYFLAPERFLGDQRASYNRDLKFKLQLVGQVAP
STSASDIILEGAGTKISLPIFGQGNGMPDQSVKEYSFRLHEHRDYQWQPSQSSRGFLSILSNLTAIKIRATYSVQGEA
ILDDVELQTAHRGAAGQPATWIEQCTCPEGYLGQFCESCAPGYRHSPARGGPFMPCIPCDCNGHAEICDSETGRCI
CQHNTAGDNCDQCARGYYGNALGGTPYDCKRCPCPNDGACMQINGDTVICTECPIGYYGARCEMCSDGFFGDP
TGLYGAVQTCKACDCNGNVDPNAVGNCNRTTGECLKCIHNTAGQHCDECLPGHFGDPLALPHGQCDRCTCYPPG
TEQNQDGITQCDQVTGQCHCKPNIIGRDCSECQPGYFNIMSGNGCENCMCDPIGSYNSTCDKFTGQCFCKPGVVG
QHCDQCEVYHYGFSQEGCKACECDESGSKGFQCDQYGQCPCNDNVEGRRCDRCKENKYDRHLGCIDCPDCYNL
VQDAANDHRAKLRNLSATLDEIARTPVTNDDEFEAKLKTVQEKVDILLTDAKYGAGESGQTYAEVLDDLHKRLN
NIRTHLVSGDELQDKANDEIEKARQNYTNAHDIIEAAKSELQTALNLLNDDGAQALAKAKNKSVEFGVQSDQISE
ISREARALADRLEAEAQVDLKNAKDANEAVEKAYELAKSAINLQQKVSDELRSEVRLELNTVKQSLNNVAQTTK
EALRKANEVYDAALTLLTDVNGLTAPEIDIKKLKKEALEANEEADRLLERVNDISNSNGKIFADFDDEYQLADVLI
LRANEQKEDDIRLLKTAQDAFDKATKAVEQGDNTLKEANNTYHTLAGFQADVQKSSEKADLALKTVPSIEQEIEN
AEQLIRQAGEALMGANKNALEAKTNAQNAQEKYAEQASKDAEIIRKKANDTKVNARKLRDEADQLNHRVQITE
SSIVKLDKAANKDDNLVDDAKRNVGQAKAGAQEAQTQIDKSIQELDDIKEELENLKDINVDDLDKLENRLDSVE
LELNRVNLTGRIEKFRDLRNAQKKWIDKYEKDLYDLEEQVANIRAIAQALPNNCYSRSRLEP

>GH20732
MKVDTATLEVISPKVQISENYIETDYEYQTSHVKRSANGKIQVHPESAALKIRTGRRVPKLGLMLVGWGGNNGST
LTAALEANRRQLQWRKRTGIQKANWFGSITQASTVLIGSDEDGKDVHLPMKDLVPMVNPDDIVVDGWDISSANI
GDAMQRAQVLDVELQDQLYKHLSTLQPRPSIYDPDFIAANQSERADNLIKGTKFEQYEQLRRDIRDFKQNSGVDT
VLVLWTANTERFCDVKEGLNSTMAELEASLKANKAEISPSTIFAMASIDEGCTYINGSPQNTFVPGLVELAEHKSV
FIAGDDFKSGQTKLKSVLVDFLVSAGIKPVSIVSYNHLGNNDGKNLSAPQQFRSKEISKSNVVDDMVESNDVLYK
PNEHPDHVVVIKYVPYVGDSKRAMDEYTSEIMMGGHNTLVIHNTCEDSLLATPLILDLVILGELCSRIQIKSNERA
DAPWVSFKPVLSLLSYLCKAPLVPRGTQVVNSLFRQRSAIENILRGCIGLPPNSHMMLEQRFDFASITNEPPTKKLK
SVSNGCATELNGKSAKVSAVASNGHYANGNANGHTNGLTH

>GJ24273
MRPEIKSFHRKQLPAFSLMLLSLMLFGFTSAQRVAPGVPPQHYQQVPVQHNQQPQYQQQQQQIHHAPNVNPQH
YQQQVQYQQVPVQQQYQQQQPQFQQQPPQQQQFQQQPPQPVQQQAPHAPHQAHHGQQQVLNAGNIQDE
RAHIQEHMQVPIDTSKMSEAELQFHYFKMHDSDNNNKLDGCELIKSLIHWHVQGAHHKADEHHGTEEEEKGKI
YSDEQLTDIVDYVLKQMDLNNDGYVDYAEYRKSEDAQSSDDKKKP

>GK25863
MGRLRHMLAVLLLVAALGSTNAESKINSPPRIIKQPPTDELLFKVAVQNKESDKPFIIECEAEGKPDPSYRWIKNGK
KFDWQAYDNRMLQQPGRGTLVITSPKDEDMGQYQCFAENEFGTATSNSVFVRKAELNAFKDEVAKTIETNEGEPF

SLSCEAPDGWPKPIVNWLIQHSLEGTIKSINNSRMTLDPEGTLWFSNVTREDASDDFFYACSATSVFRNEYKIGNK
VLLDVKQTGISAALNKQQPKRQYVTRKNEVALRGKRAELYCIFGGTPLPQTVWSKNGAPIEWSDRITQGHYGKSL
VIRQANFEDEGSYTCDVSNGVGNAQSYSINLKILATPYFTKEPEVQNAAEDEEVTFECAAAGNPEPTIQWIHNGKP
IAQSPPNPRRTVQSNRIIIRDLVKADTGNYGCNATNSLGYVYKDVYLNVLALPPEIQEAPRKEATVDGKNVTLRCR
VFGAPKPQVKWIHNHQELTGGRYNTLPSGDLEIQEVAFSDEGDYTCYATNKFGSISANGTLLVMKRTSIIHEPQNY
EVAAGKSATFRCNEEHDEKLDLVIEWWKDGQAIDFESEARFMKTNDNSLTIPKTVELDSGEYTCVARTELDEASA
KANLIVQDVPNAPRLVGIVCMANRIRVTWEPQGDNRSPILYYTIEFNTSFTPDSWDISYDKVPSTETAFVVDLTPWS
NYTFRVKAFNKIGPSPPSDHSEFCTTPPDVPYKNPDNVQGMGTEPNNLVISWTPMPEIDHNAPDFHYRVSWKRDIP
AASWESKDIYDWQQNNLVIPDQPTYVPYLIKVVAINKHGEANVAPTEVVGYSGEDRPLEAPTNFSMVQVTAATTA
MLRWNHVSPESVRGRFKGYKIQTWTEKEGEEALREIHVDAISDQALVTQFKPDSKNFARVLAYNGRFNGPPSVVI
DFDTPEGVPSPVQSLDAYPLGSSAFWLVWKKPLQPNGKLTGYKIYYEEVKGSYVSERRELDPHINDPLITSAKLAG
LKANTKYRISITATTKMGEGSEHFIEKRTLPEDSQMPAVPGFIVQQLKSENNNAKYRVNWQPSTEGHAGTHFYTQ
YRIKGEPNFKTEKPEFSKDYQEIGGLNPDNVYEFRVVSVDGDHETPSELREIVIDGPTKVPNQNVANAGWFIGMM
LALAFIILLFIIICIIRRNRGGKYDVHDRELANGRRDYPDEGGFHEYSQPLDNKSAGQQSVHSAKQPGVESDTDSM
AEYGDGDTAQFTEDGSFIGQYVPGKLQPPVSPQPINNTPAAHQSPTAPPAPTQAATSSNTPAVATYV

> GJ18745
MKSNFLILLLVVPALICTFHAPHRFAAFAEGSAEDDLVDVEGEDGAVTGDDAENEEDADASVTTSPYADTYLLFTK
PLYTAGQQLDLPGGKPVEFLIGFTNKGKNEFVIETVEASFRYPMDFNYHIQNFSAVAYNREVKPGSEATVAYSFMPS
DTFAGRPFGLNIALNYRDGAGAQFTEAVFNETVLISEVDEGLDGETFFLYVLLAGIVVLLLVIGQQYLLGSSGKRK
RAAAKKTIETGTANDSNIEYEWLPQETLRVLQQSPKSKTPKTSPKPGKQSSPKQRKVKRGDD

> GD11983
MSHLGIFFLLLAFVLVVNSIPAYAGVAHAHKHEKHNSKERLKDGIYAGRDAHHHEDGDHNVEFDHEAIIGSLKEA
KEFDKLTPEESKKRLLVLIKLMDLNKDGFVERHELKAWILRSFKKLGEEEAADRLEEIDVDGDGKVTWKEYLKD
TFEMENEDEKKELIDFDSYDEEKEMIKSDKELFNAADLNKDGALDSKEYVLFYSPEEHPEMLPIVLEHTLREKDT
NHNGEIDYAEFIGSAGQDRSREWLVEEKDRFDHLDENEDGLLTGNEVLNWVVPNSEAIATDEVDHLFVSTDEDH
DDRLSYLEILNNYDTFVGSEATDYGDHLENINHFLDEL

>GG17054
MSQNVVGVFLVLIAVANAVKVPVTIYYESLCPDSAKFITEQVFPAVKGELRDYVEINWVPYGKSHFTTQGAEVIFD
CHHGPNECYGNKVHACAIEHIQANSYQIEFTRESLTLDFINCMMKAGKNFDDNVYPGARCARENHISAWENIQQ
CANTTEGSILLKKQGEATMQFQNPLTSVPTVVFKQQYDAKENDQAMSSFLNVVCKYIPQPQPKVCAALNSAVATT
VTPLLAALAYLLVRFF

>GH13810
MKSFSLFAVLLLALAAFATLTQASFCPCDLTQKGQICGSNGITYKNRCEFECTQKDYKKLGRTLNIAKTGPC

>GI22092
MEIFKRLNFLLVLFLNYKFSLGYLEYDNWVNIELSHAIDPKSPNSFTYRGNVTISSLDIGLAKSNQEALTGIQIQNLQ
TLARMDKLYRLKAEVSYADGKKQEFLSSTKACNLIMSNLNDILWISIDPNGYINAATVLTSRSDICFCENISVSGLE
DFNTDVLIRHTETAPVPDTTSFIQKLEREREARERGDVRDNRGFFSKYWMYIVPVVLLVFISGATNQDNSK

>GE26334

MFRKVLFATPATAVLLAAVPVKQSEVKAAENVEANTNFTCKPSDLPLYQSLHELHSTKKESHPPKDSPTRQTIESGI
RVVRTEIQSGLNVLAEQKAHFDHYYETGKAHTQSTIDYLNEPQNILPRSGAIAVGGLSGFIFAARGGFIKKVLYTTI
GAGTVASLCYPKQAEVFARDALVQARKGYAIAYNFVKGVKPGDEVTPEPVSKFPTSMEDLKYLALDLYDEAKEA
IFGKKQ

>Chitinase-like protein Idgf4
MKFVILLALSLAVLASAQHASNSHLVCYYDGSSYVREGLSKLHTNDLEPALQFCTHLVYGYASISPTSNKLVSSND
KLDLDIGSGLYRTVTGYKKKYPHLKVLLSVGGDRDEVDPDNNKYLTLLESTNARIPFINSAHSLVKTYGFDGLDL
AWQFPKNKAKKVHSGIGKLWKGFKKIFSGDFVVDEKAEEHKEEFTALVRELKNAFRPDGYLLGLSVLPNVNSSLF
YDVPAIVNNLDYVNLHAYDFQTPERNPEVADFPAPIYELNERNPEANINFQTQYWLNNHCPATKINVAVAAYGRA
WKMTKDSGLTGVPPVVETDGVAPAGTQTQKPGLLSWPEVCGKLPNPANQHLKGADGPLRKVGDPTKRFGNYAY
RSADDSGENGIWVGYDDPDTAANKAAFVKARGLGGIALVDLSFDDFRGACTGDKYPILRAIKFKL

>GF23312
MSLLKVCTFLAAISLAVADIVDFKPCGDVDACTIHEVRIDPCPQASEHLACHIRRRRPATMSFDFTTNFAAETLEAG
LAWQKSATQDLPLITMDKQACKYTPCPTVANQRQTYTMDVPIESKFPLNPYTIKWTLKAPTGQVCCFTVDIKVVR

>GJ19442
MKLSSMAKTLLSIAFFTIVINCVLGSSSSSGDIDLSQINVNDLKQNLPPEFANTNLTLDDVKTLVRNKCIEVAGKEE
GEAAYTEIENAVPTLTECASNILNFTALQAEIEKASPDGELDVVFNKYCNKQPEALNCVSDFNNKLAACLDKEEKE
HQDVFMRIIKSLLSFICHKGGDQIALFIAEKGPECLDSKKDDIQNCINSTLSSYIPKNGFDDIKSLPKFVMGMKQCR
EMEELEKCIVQKLETCSEITPANIMESMFRFIKNETICQNAPRVAKQASITSGVNSSAITGGVIWLIAAHLLYQIIKSR
VQT

>GK12751
MKIFMHVVLVFLLLQMHLAIGDKKQSKKYSREANEQQYNTQQKQHKSKEKSYEGEFKLTTERYDPDFRNLQRPF
RMAKLNLVWAKGTKCKLTEPKLKSLYMELKIQDKEEIAWKQLSSQHKDKEGIKEAELRQKLVGIMSTYDLLEHFE
DTQDKDKVKPYKKFYDPEDSHLNKSAFKDKKLNRLWAKAEVAGFTKEELDSLKEEFQHHQDKVDVYYSLLEDI
GSTNGPKHENAVNEDELDIFNMVANDPNENEINTPEMNLKKYESDINKLRDHHHGIKDHYERLERLVSSGPHSQE
FVEPKVQGLWRVAQASNFTEKELSSIKYELHHFESRLLKLRNLHAEHAMQKEKYKNEKQKDKSGRFEDMEDHIK
KQTRKVEKIQDTIEKTIFRHSEL

>GL12691
MKLFAIILTLALYAFALAQSLANVKCQMNCDLQDFRPVCGIDDAGKTKTFNNLCILKTENCLRHLSFQKTSDGVCP

>GI15820
MRNFVNYVVIVLSLCTVGSLAIRCYQCSSDQDRKGYDSCGAYKPFNRTEHIPIECNSDESHMPGSFCMKVVQQGP
RGFIWDGRWRQVIRRCASVSETGVTGVCNWGVYENGVYWEECYCSNDSCNAASSISLSKKSLALAIIAILA

>odorant binding protein 1
MKYIVAVLLAALVAMAAAEEYKIRNQDDLLKARKECMEAKKVPTEHIEKFKKFEFPDDEVTRCYIECIFNKFQLFS
PTEGFKTQNLIAQLGQNKENKDAVKADIEKCADKNEQKSDSCTWAYRGFKCFISKNLPLVQESLKKN

>GF24261

MLSKLCILILCAVLTAGNKYAESGSCHSFAGGSVYPSEGPRGDHNLQTTKAVISKSAPTFEGTAVVKGEFVKLSLSQ
YHGKYLVLLFYPLDFTFVCPTEIIAFSDRIEEFRKINAEVVAISVDSHFTHLAWINTPRKEGGLGYVKIPLLSDLTHKI
SRDYGVYLEDLGHTLRGLFIIDHRGILRQITMNDLPVGRSVDETLRLVQAFQYTDTHGEVCPAGWKPGAETIVPNP
KEKTKYFEKINK

>AGAP011367-PA
MKCFLVFPLLFVLLCVNAYDFDDSAFNEYLFKELQSHYEEDEVSHRTRREATDANECSKRNWKKDMQCCKGGN
VNGDQLELFKSVKKQCIADLKGEPADDAVDPFDCEKMQQVKEKMICITECVAKSFKSLDEHGELQRDAILEGLRG
QIGTVQWKLDAIEGYVDTCLAEVKEKREQKQKAGELKEEGCSRSPLAFHSCMWRQFWNGCPADLRVDSPKCNK
LRERVANGDTRFFGKHFLHKYYPNPHDEE

>GF24406
MWKRKLLILVKVFLLMTMLASATQANSDAGKTIKTLTPNANIAREQRPLLANSKIKVHIECEGRDGLDVNIEWQL
YERQCWTDKRMYDIQKYDIEENLPMQNKSEFGFITMDSKTTECSTHPILLSEKQWPVQETTASGTAALSPETAAL
ASVKKANNTKKHPTAPLDKNIITIGRDGIYELTLKIEAVKSSEPVDFSAHVQLDIVGPDGGYLSAIDHPLLAFYGIM
CVVYVIFGIIWLFVSFMQWRDLLRIQFWIGGVILLGMLEKAFFYAQYYSLNATGVPVEGAELMAEFVSCAKRTLA
RMLVIIMSLGFGIVKPRLGPMLHRVVGVGALYFVLACVESYLRVTSTKTDEQLVAAIPLAVLDTGICWWIFTSLVQ
TTRTLRLRRNMVKLSLYRHFTNTLIFSVLASVIFMLYALHVRKSQNCTPIWRNIWFDAAFWHILFSVLLLVIMILWR
PTNNNQRYAFTPLLDAPDDEDDDEEDQFVADAYGVKMRSSHANGGTKTPPNAQRGTTTEEDDLRWVEENIPSSM
SEPALPVLDSDEEIINTRFEVSKMQ

>GF13366
MTSRFYKFGVTALSACIGSIAATWVCTDRNTSAYVVHNEIVRPPKRKRTLPPRPDQMKSLQSGEEYDVLIIGGGAT
GAGCALDAVTRGLKTALVEADDFASGTSSRSTKLIHGGVRYLQKAILGLDFEQYRMVKEALQERASMLESAPHL
AHPLPIMLPVYQWWQVPYYWVGIKCYDLVAGDRNVKSSYYLSKKDALELFPMLKKDKLCGAIVYYDGQQDDA
RMCLAVALTAARHGATVCNHVEVKELLKKDENGKKVLCGAKVKDHISGKEFIVKAKCIINATGPFTDFIRKMDDP
NVKTICCPSSGVHIVLPGYYSPDQMGLLDPSTSDGRVIFFLPWQRQTIAGTTDLPCDITHNPSPTEDEIQFILNEIKN
YLNTDVEVRRGDVLSAWSGIRPLVSDPNKEDTQSLARNHIVHVSPSNLVTIAGGKWTTYRAMAEHTIDAAIKACN
LKPERPEAVTSYLKIEGGQGWTPTMYIRLVQDFGLECEVAQHLAKSYGDRAFAVSKMASLTGKRWPIIGNRIHPEF
PYIDAEIRYGVREYACTAVDMIARRLRLAFLNVQAASEALPVIVDLMGEELHWSKDEKEKQIKLANEFLAHEMGQ
MVNRTSKERIPIKLSKDEIQTYVKRFQLIDKDKKGYVSINDIRRALKSFGDADVSGEQLHEILREIDTNMNGQVEL
DEYLQMMSAIKTGDVAYSRFARMAELEEQKHEAAQLKQKISVDRSGGGL

>GK24231
MQSTILASVLVILLAVQQGAAIKCYVCNSHKDANCALDIPPEHLLKDCEEDYSTRGKGIPTYCRKISQIIEFSVNNL
PPDSRVIRSCGFLNQTSTNYCYQRAGFGGRQVVCSCDKDNCNGAGALNLSLGVMFAAAAASGW

>GF23809
MFTLWTLVEASLLCLNAVCVLHEERFLAKFGWGAQGSNMQEFGQPTAKAQILNLVRSIRTVAKIPLIFLNVLAILIK
LILG

>serine protease inhibitor 1
MTSLRSCFLVIQTLLAATLINMATAGTIAPSLAASRNLFAADIFQALAQTRTQQNVVFSPASVQSCLALAFLGAEGE
TAQQLRSGLRLPAALDKPSIAADYAAFLQKNFHKDANAAEDAPKLRMANRVYVNESLELSAKFNELAKTSFESEA

VPTKFADAANAVQTINTWVEHETEGKIKNLLQPDAVNAETSAILVNAIYFKAKWLHPFSAFSTSDHEFRMSDGQT
SSVPMMYGDERVKYGELADLDAKAIELPYKNSDLSMLVLLPNKVDGLAALEQKLSNADLNLIVERMRGADVDIF
LPKFRIEFEVDLKQPLQQLGMVDMFSGSADFSSLFASGPQQRVDDVKHKAFLDVNEAGSEAAAATFMKIVPMSL
NLDQKVFKADHPFVFAIRNKEAVYFVGHVAR

>GH21921
MFYNILLLLLASYQAAVALENGLARTPPMGWMHWERFRCITDCQKYPNECISENLFKRTTDLLVSEGYAAAGYEY
VIIDDCWLEKNRDNATNKLVEDRYRFPSGLNHLADYIHEAGLKFGLYQDYGTHTCAGYPGVINHMELDAQTFAE
WDVDYVKLDGCYADTTTMDAGYPEFGRLLNQTGRPMVYSCSWPVYQEYEGKIPDYEALKQHCNLWRNYDDID
DSFESVAKIMDYFSKNQDRIQPHGGPGHWNDPDMLILGNFGLSYDQSKLQMAVWAILAAPLIMSNDLATVRPEIK
AILQNREIIAVNQDPLGIQGRRVLIQKNVEVWTRPITPRNSAGEYSYAVAFVNRRTDGAPYPISFNLKEMGLDSLRG
YDVVNLFEKSQKLGVIKQTHIFNTRIIPNGVNFFKFTVL

>GA12379
MKFSINMCSRNIKISVVLFLVLIPLITALPHNNHNLSKRSNFFDLECKGIFNKTMFFRLDRICEDCYQLFRETSIHRLC
KQNCFTHETFGECLKVLMIPEEEVSQLQYFVKVINGSPMPFAFTNMQ

>Gmfb8
MKQIFIFIASALLLLQSSHAFPRTKRNTEAVLALFQNSSSEEVDPKVQLAVTTLNDIVQISKWVLEKGSVVVNNAV
KELEALPVQDELLRANITRMSKIGTESAQFKLEEDEQSILKLLDYMVSFTEMMIDYENMPDDSKQKAVLKTALEN
NGYNKLEKDYEEKGVQLAKDFDQEFGKYVSGLTPAEKVDEAKLLKWYDDYKAETDEDKKFDKFGDFF

>GA15794
MSRKTLICSIVLLCLLSGNHGVFALFEGCDSTFQINPGTTYLQSPYYPNTYPPWTSCRYQIIAPVDYTIEANCTIDLP
SNAGQCTTEHFYVSTEGDPQLRDSEQFCGQGTFTRVSLFRKLTLAYASYGNTGRFLCSLTIRPQQCECGWSTSTKI
ANGQEVGTNEFPFMVGLQNLASSTDIFCGGTIISHFHIITAAHCARIQPDASQIIAHVGFTQISTLNNSRYAAAYRIRS
ITIHPGYTDDPPVNDISVLVTNILIEWERGVGPICLPPAGSSTFAYQTVDVAGWGTESFAGPTTDALMKANLMVVE
NSVCQEKYDVPIYSSQICTTDYSGQGRDACQYDSGGPVVWRSSRMFLVGCISFGQACGQPYGTGVNTRITYFLN
WIQQVTGYTTCVKPLG

>gasp, isoform A
MNKFLFVFVALFGAAVAQDNFKCPDDFGFYPHDVSCDKYWKCDNNVAELKTCGNGLAFDASDPKFLTENCDYL
HNVDCGDRSQLEPPISTPHCSRLYGIFPDENKCDVFWNCWNGEPSRYQCSPGLAYDRDARVCMWADQVPECKNE
EVANGFSCPAAGELANAGSFSRHAHPEDCRKYYICLEGVAREYGCPIGTVFKIGDSDGSGNCEDPEDVPGCEDYY
GDLDLKSIRKSELLAGLNSEGRTKNAPKTKAAAASS

>obstructor-E, isoform A
MFKILLSATLCVAMFGSFAHAQCQQPNGTQAVSGSCDAYIECKNGVAEEKLCPDGLLYNEKSSGYPCGYPIDVECS
QGQARTQPA

>CG31195
MLSAKSSILVRSYTLFLLTLVVGVFAQFEWQQRDAFDEIKYRFDKVNGDNCPIQHVSDLYLPEDTVSHKPDIKEINI
NPIFPNRTALLHLHNMAMSRSFFWSYILQSRFIRPAINDTYDPGMMYYFLSTVADVSANPYINASAIYFAPNSSYTS
SYRGFFNKTFPRFGPRTFRLDDFNDPIHLQKISTWNTFDVQDLGAHLPDTKSKDYTSDLYKINEWYKTWLPDDVE

GRHDTKTTYQVEIRYSNNTNETFTFHGPPGADESPGPVKWTRPYFDCGRSNKWLVSAVVPIADIYPRHTQFRHIEY
PKYTAIAVLEMDFERLDINQCPKGEGNKGPNHFADTARCKKDTTECEPLHGWGFRRGGYQCRCKPGYRLPNVVR
RPYLGEIVERASAEQYYNDYDCLKIGWVQKVPIQWERESYDVREKYLDLHPEYRNYSTGSRALHEEHINIDQVL
KFIHGVNYRTCKNFHPQDLILRGDVGYGAKEQFENEAKMAVRLANFVSAFLQISDPNEVYTGKRVADKPLTEDQ
MIGETLAIVLGDTKVWSASMLWERNKFTNRTYFAPHAYKTELNTRKFKVEDLARLNKTHEIYTEKKYFKFLKQR
WSTNFDDLETFYLKMKLRHNETGEFMQKYEHYPNSYRAASLKHGYWAQPQFDCNGYVKKWLVTYAAPFFGW
DSLKVKLEFKGVVAVSMDMLQLDINQCPDSYYEPNAFKNTHKCDDLTSYCVPIMGRGYETGGYKCECLQGYEYP
FEDLITYYDGQLVEAEYQNIVADKQSRFDTFHCRLAGASGLQVATSLLVSLLGLTLVLMYKFR

>GA19485
MDVGKYLGLLALMLLSLLALYSSPVEACSCMPSHPQTHFCNADYVVIIRVMRKSLRLVQDNVAYKVEVKKSYK
MNEAGHKLLKHGRIVTPNSDAMCGINLDIGKLYVIAGRGPYLNSCSYVKEYLKMSVVERRGFAGAYRKGCKCSV
QPCFGQSCLSEHQSATSCKWSPFAKCETDYSACIPSHYRTPEGIISKCHWRRTPAYKKCMSDP

>GH20023
MRLLLLFALVSLVPKTLTKRAEDDKKPEWAKKDIRDFTDADMERLLDQWEEDEEPLEPDELPEHLRPQPKIDLSN
LDSKNPEDLLKASKKGRTLMTFVSVGGDPTREEADTITKLWQTSLWNNHIQAERYMVDDNRAIFLFKDGSQAWD
AKDFLIKQTRCKGVTIENKEYPGEWS

>cellular repressor of E1A-stimulated genes, isoform A
MSQAIAIISGLLFALLAVDSCAGYSAHGRQPSGLSDAALARRLVHQANWAAVGSISTHEKIIDFPMVNIMAVDDSA
LNGSSTGHIRFFLLNLDFTGRDTQQKNKVTFFFTDDQNGACQRRGVDPMEPACPRVMLSGSVKKLDAEDPSYND
QLQAFYNRHKSAEKWAAHSHNFDFSELEIENIFIVAYQGGPRVIPAEDYYNATI

> juvenile hormone epoxide hydrolase 2
MGTLLRITFVVLAIVVGLCVHKYQELTSSAPIPQLNDAEYWGPGSAAKYKENTAVKAFDISAKPELIEDLKTQLSR
PLVLTEPLEGVGFQYGFNSKYLKEVVAYWRDTYLPKWGEREAFLKQFPHFETQIQGLRIHFIHVKPKSTEGKKVVP
LLLIHGWPGSVREFYRLIPLLTKPNPKSEYVFEVIAPSLPGYGWSQGASKVNFGPAQMSLVLRNLMLRLGHEKFLI
QGGDWGSILGANIVTLSPQNVLGYHSNMCTTSHQMIHLHKLLRNWFPSFFIKEDNSIFFKPFGKELSYLLEESGYM
HIQASKPDTIGTTLTQNPVGLAAYILEKFSTWTNPAYRQLEDGGLTKRFTLDELLDNIMIYYTTNSITTSQRLYSEGF
NFAYFAMNLDGTHINVPTGCARFLHDLLHLTDSELGLKFKNIVHSTYHKEGGHFAAMEVPHILYSDFVEFVAKVF
RKPQPKQ

>GA20648
MRFAQYICFVAISLAMVPACLGLLDGIYCGREDCYDVLGVTRQSTKSEIGKAYRLLARKHHPDMHRGVEAKAEA
EIKFKLVATAYEILRDDDSRRDYDYMLDHPEEYYSHYYRYYRRRVAPKVDVRIVIVATLTIVSIIQYYSGLQRYDSAI
KYLATVPKYRNQAIEIAKEEIEKISNRKGKNRMSKTDQREEIERIVRKVIEEKMDVRGGYAKPSIYDILWVQLLICP
YTLLSFLVWNAKWIWKFTIRKQPYGRDEKLYLIRRYMKMGENQFNGLEDHQIEEYLRLELWEKDKFEEWHEEQE
EEMKKKLAENPRYKSYRRYMKNHGPGRMTFED

>maltase A5, isoform A
MIKRLLLFLLVSICSGVTVVSAGGFPNYHAHFDFIAWDKHSVAAKVNASQKWWQSAAFYQIYPRSFKDSNGDGV
GDLKGIAEQLPYLKEIGIAA

> insect-derived growth factor
MFRILRLALVACLCSVVLARFHTRSQLAASHMAAYAYRPDGNARNNRPTPEAYNAERSTFFRYEESRGLGAEIELN
DRELLANQIIMAAKTYEYEEGIVTPHLFKPSKHFFSVLEDIAKTPLFGYLQAMPKGGVLHAHDTAMCSTDFLITLT
YRENLWACESSGSLEVVSLRFARTKPMLAVNGKDCVWTLLAEIRARHGAAAVDDFLREHLTLYPKEKFLDNNEA
WASFMEIFSLLDGMITYAPVWADYYYNALKEFRADGVQYLEFRSTLPQLYDLDDGEYTELDTVRIYKETLEKFM
KEYPDFIGSKMIYAPIRNASPETVEQYIRICVEIKATYPDFVAGFDLVGQEELGRPLKDFIPQLLSVPADIDFYFHAGE
TNWYGAAVDENLIDAVLLGTKRIGHGFALTKHPLVLQALKERNIPVEINPISNQVLQLVSDYRNHPCSHLFSDNFT
VVISSDDPSFWRATPLSHDFYIAFLGIASAHADMRLLKKLAINSLVYSALNEQQKEIALQKWQLQWDEFIEKIIQG
DTDGAAGRLQTNRID

> cathepsin F like protease
MRLAVGATIVIVALSLLSVLVTGQEAGAADEVHARAKRSSNLVGGAKTLDKKDAEQRLQASLDKLAAGDGPNYR
ISKVYSATTQVVSGTLTKIDVELFNENDEKERCTVKIWAQPWLPNGYEVTFDCPNKELVKRRHSRSVEQLEKKTH
KKRNHHSLDKTEHLFSKFQIKYNRRYHTAMEHQMRLRIFKQNLEIIKELNRNEMGSAKYGITEFADLTSTEYKQRT
GLWQRSEDKPSKNAPAVIPNIELPKEFDWREKGVISKVKNQGMCGSCWAFSVTGNVEGLHAVKTGKLEEYSEQE
LLDCDTSDSACNGGLMDNAYEAIEHIGGLELESDYPYEGRKDQCKFSKSKVHVKVKGGVDLPKNETAIAQWLIA
NGPISIGINANAMQFYRGGVSHPWKALCSKKNLDHGVLIVGYGVSDYPMFKKTLPYWIVKNSWGPKWGEQGYY
RVYRGDNTCGVSEMASSAVLE

>GK15467
MAVQFQRCLRVLLLIALISAVSSAPQQKQTAQKSVAVVSGPAGGGPPRVQDPKCLQPKEPGPCRMNLDRYYYNSQ
TNACELFKFGGCRGNDNKFGFLKTCEDACVTPK

>putative zinc-metalloproteinase precursor
MRTCLSLVMLLVISTSRAIPVDSVREEDPELTAGYFEGDIVLDAAPRNGMSNTSLYWPNGIVYYKIWEGFFDLVQE
TVIKGAMKTIEDVSCIRYILADDDQPYFVNITGNPGGCYSTVGFTDDIQKYNLEPFPINEGCFRIGTMIHEMLHTLG
FYHMQSTFNRDEYVQIVSENINPQYLHNFNKYDKDMVEDFGEAYDYGSILHYSPIAFSANGEKTIEPLQEIPVGLM
GQRVTLSEGDIRKLNKMYNC

>Gp150
MGNQHALVLLVLLALGAVQQTFTSSLHAADLTEEKYNLLGDVDKEEVAKLVHSKEHGIKISTPKVKEIPITSTTTTT
ETTKQAKKLAATTQHQELGGELNNALYEDNESAEQQQMSDETLNDLIEASKHSVALADDPRFNKMLPHKLKTEN
ATATSNKSEENYYDYDTDDYDDDYNYDDEVAATESTQKAKSGSSTTGKPNRAVNTAAKNSKKVEMKPIKEEKTS
SSKLKDDTQIVDNKQQQSGKKIEDLDEDDYDEDDDDDGDEDDINDEEEIVYNQNVPCPRYCVCARNVNSYLVAT
CSRFDPETQKFGSDITDLIVTDVGPKYPILLGPEFFVQMGLKYVSSIKISNCTIEFLHSDAFHGLDELYSVNLTNVGL
TVINPNTFSKNKKLRLLTISGNDLSVMSNVHFLLNSSSIEELDLSRNNLMELHPQAFSKLYNVVYINLSQNNLEKIP
DHVFDSVETIEELDLSYNALKTLPANVFNRTTLAILHLKYNSITGDLHFGNDDLQQLDLSFNNIKHIHHGMFDRMT
GLTNLNLKGNGITKIQADSFLALKRLRHIDLSINDLDQVSSMIFYKNSELDVIRLNDNPRLSQLPTDGFLSYNGLFT
VYFLDISNCAIGALGHKTFSTMPHLATLKLAWNNINNLERDTFTSLNKLTELDLSNNLIARLDELLFMNNNELTKL
NLAGNPIRKLSVRLFMPLAKLQELDVSDCELSSLLSENQYGMGRKYKFYETLRSFNASSNQIKRISSGDVRSFKNL
HTLDIAHNPLKCSEDFQDFISYVSLRPYLGPNKLPTHAELEGGDASLVEYQPHADWAALAHEVCRHQEEGTITKP
TQKSLEKRIEQADKELDSDAKSLWKIVENTKTYKHKILKDTEKSDVVIAGNLDDESAEEDENTAEEDKDEEDEAD
EN

>GJ17158
MFSHINRCLLLLCAITVTITTSSATVLATTRSTAKISPEIINELKDFLDLIPTATVDEIVAKHYITDSNFREVFKYLRGT
QFTALLQQAQQIPEVIDVLDYLHLLVPNAQQQPAYGRADAGSSEVQLSSEPQLSFVLLNDLAEENEVLPRAMDEG
TVSTFSPPILPPSARHLRTFNSFVEELLGHLPHDRYVQLINQKRQQNLAFAQFYAALRSAELRPMVDATLKSSNLT

> GJ23716
MTGTRFARTGRCRFFVCLAVLTAMLCMVAIFHSSQQQLDETREQRLRCERQQEQLNEQLQNMVEQKYNQQKTLE
QERNEQMEARHNLEQKLKELTILRTNEQMDIHLRYDKLQQAHKLLKSEHNELVTECQKSKKELMENTNALEKKL
QSVRSEADKEKNVLNGEITLWREKYNSIVAEKERLEILLDASGKTAELQSKVINLEHIIAQYEQHCDYKPETKSNN
EKPLNPPNTLRNTSPRTRIEKSFNEQVPILPKPIEKVADGAESDGKVLNQLRQPPANQSDASSNNKLILPLSAPRKAA
TTEASKSEQHVLEVNAPIKEINNTSTTSSAFPPLPMPPNPRKLPENVAPIPANFEELLKEDNANLKADLAKEADVNA
AEEVQKHGNDGAKNNRYANAINEMEPKASNKEKVIAAANAANEDSGAHEIKDNEFINDQNFNLPANEENNFFDG
GVKNEPAGNVEEKPKGDDNDDDEGLAGAVDAGDIAVKNHNLLDTNNLNNEVAGDQGKEFPDELRLEENNEEDE
DEDDYSNPGARQQGEQAIRN

>putative salivary trypsin
MAYKLAVLTVCLLSLWISTTQAQATPNIDDLLNKIFTTEQTPFQPQPTKTTVLKPDPQPNPAPQPDPGSGKSCGVNK
ECVARFLCGEDGSVIRDGQTLIDIRFDGDDVCTYLEVCCNTGDKLTEPDVPIKPVETHEGCGYRNPSGVGFRITGD
KEGESQFGEFPWMVAILRDEVIGGETLNVYECGGAVIAPTVVLTATHCVLNAQPELLIARAGEWDTQTKQEVLPH
QDARVKEIIRHENYNKGALYNDVALLILATPFTWEENVRPICLPEPGVNFDHSRCFATGWGKDKFGKDGTYQLILK
KIDLPAVPHDTCQAELRKTRLGKFFNLDKSFMCAGGERGKDTCKGDGGSPLVCPDPNNSKRYYQAGIVAWGIGC
GEENVPGVYANVPYLRNWINEKLAARGIDFRYFTA

>GJ18156
MKILIVALFATLALQNALASVKEEITATEYIENLNKEIARRTNLETEASWAYASNINAENERKKNEVSAEVAKFMKE
VSTDLQKFNWQSFKSENLRRQFKMLTKLGYAALSEADYAEYLDVMSTMESNFAKVRVCDYKNATKCDLSLDPEI
EDIITTSRDPEELKYYWREFYDKAGTAVRTPFEKYVELNTKAAKLNNFNSGAELWLDEYEDDSIEKQLEDIFDELR
PLYQQIHGYVRYRLRQHYGDAVVPEKGPIPMHLLGNMWAQQWSSVADIVSPFPDKPLIDVTNEMVAQGYTPLKM
FQLGDDFFQSMNLTKLPQDFWDKSILVKPDDGRDLICHASAWDFYIIDDVRIKQCTRVTMDQFFTVHHELGHIQY
FLQYQHLPFIYRAGANPGFHEAVGDVLSLSVSTPKHLERVGLLKNYQRDDEARINQLFLAALDKIVFLPFAFTMD
KYRWALFRGEVDKKDWNCAFWKLREEYSGIEPPVVRSEKDFDAPAKYHVSADVEYLRYLVSFIIQFQFYKSACIK
AGQYDPTNPELPLDNCDIYGSFEAGQAFKNMLSLGASKPWPDVLEAFNGERTMSGKAIAEYFEPLRVWLEAENK
KNNVPIGWTTSDKCVSS
